# NSAIDs and Cancer Resolution: New Paradigms beyond Cyclooxygenase

**DOI:** 10.3390/ijms23031432

**Published:** 2022-01-27

**Authors:** Oluwafunke R. Kolawole, Khosrow Kashfi

**Affiliations:** 1 Department of Molecular, Cellular and Biomedical Sciences, Sophie Davis School of Biomedical Education, City University of New York School of Medicine, New York, NY 10031, USA; okolawo001@citymail.cuny.edu; 2 Graduate Program in Biology, City University of New York Graduate Center, New York, NY 10091, USA

**Keywords:** NSAIDs, aspirin, cyclooxygenase, non-Cox targets, resolution, cancer

## Abstract

Acute inflammation or resolved inflammation is an adaptive host defense mechanism and is self-limiting, which returns the body to a state of homeostasis. However, unresolved, uncontrolled, or chronic inflammation may lead to various maladies, including cancer. Important evidence that links inflammation and cancer is that nonsteroidal anti-inflammatory drugs (NSAIDs), such as aspirin, reduce the risk and mortality from many cancers. The fact that NSAIDs inhibit the eicosanoid pathway prompted mechanistic drug developmental work focusing on cyclooxygenase (COX) and its products. The increased prostaglandin E2 levels and the overexpression of COX-2 in the colon and many other cancers provided the rationale for clinical trials with COX-2 inhibitors for cancer prevention or treatment. However, NSAIDs do not require the presence of COX-2 to prevent cancer. In this review, we highlight the effects of NSAIDs and selective COX-2 inhibitors (COXIBs) on targets beyond COX-2 that have shown to be important against many cancers. Finally, we hone in on specialized pro-resolving mediators (SPMs) that are biosynthesized locally and, in a time, -dependent manner to promote the resolution of inflammation and subsequent tissue healing. Different classes of SPMs are reviewed, highlighting aspirin’s potential in triggering the production of these resolution-promoting mediators (resolvins, lipoxins, protectins, and maresins), which show promise in inhibiting cancer growth and metastasis.

## 1. Introduction

Inflammation as a fundamental response to injury has been recognized for thousands of years. The Greek physician, Hippocrates, may have been the first to regard inflammation as the beginning of a healing process, introducing words such as *edema* and *erysipelas* to describe its symptoms. The first comprehensive description of inflammatory symptoms can be found in *De Medicina*, written by Aulus Celsus (~25 BC-AD 38), who described the four symptoms of inflammation as *rubor, tumor, color, and dolor* (redness, swelling, heat, and pain) [1]. The fifth sign of inflammation, *functio laesa* (impaired function), was added by Galen of Pergamon some 100 years later [1,2]. The relationship between inflammation and cancer was first proposed by Rudolf Virchow in 1863, who noted the presence of leukocytes in neoplastic tissues [2,3]. 

Inflammation is the body’s response to an insult, such as infections or injuries. Inflammation can be acute or chronic. Acute inflammation or resolved inflammation is an adaptive host defense mechanism and is self-limiting, which returns the body to a state of homeostasis [4]. However, unresolved, uncontrolled, or chronic inflammation may lead to various ailments, including cancer [4,5,6,7]. 

Key features of cancer-related inflammation are infiltration of white blood cells, tumor-associated macrophages (TAM), cytokines, such as IL-1, IL-6, and TNF-α, some chemokines, cell cycle acceleration, cell proliferation, evasion from apoptosis, and stimulation of tumor angiogenesis [4,5]. Important evidence that links inflammation and cancer is that nonsteroidal anti-inflammatory drugs (NSAIDs), such as aspirin, reduce the risk and mortality from certain cancers, such as colon [8,9,10], ovarian [11,12], prostate [13], hepatocellular [14], skin [15,16], esophageal [17], pancreatic [18], breast [19,20], bladder [21,22], and head and neck cancer [23]. Recent reports indicate that daily aspirin use, whether regular strength or low dose, resulted in reductions in cancer incidence and mortality [24] and also prevented distant metastasis [25].

NSAIDs have been in widespread use since the advent of aspirin by Bayer in 1898 and COX-2 selective NSAIDs (COXIBs) since 1999 [26]. Aspirin is the only NSAID that covalently modifies the COX enzymes. It acetylates the catalytic subunits of the COX enzymes by accessing the enzymatic active site via the arachidonic acid (AA)-binding channel [26,27]. Aspirin acetylates serine 530 of the cyclooxygenase-1 (COX-1, constitutive) enzyme [26]; the interposition of the bulky acetyl residues prevents AA from accessing and binding to the enzymatic active site. This inhibition of AA binding blocks COX-1 from synthesizing prostaglandins (PGs) [27]. This modification also occurs with the second COX isoform, aspirin acetylates serine 516 of COX-2 (inducible enzyme), inhibiting AA binding to COX-2, and thus COX-2 activity [26,27]. The acetylation of COX-1 and COX-2 makes aspirin an irreversible inhibitor of the COX enzyme [27,28], which lends aspirin its various properties, beyond anti-inflammation, antipyretic, and pain relief. Blood platelets contain only COX-1 [26]; thus, when AA activates COX-1, thromboxane TXA_2_ is produced. A vasoconstrictor, TxA_2,_ also aids in the aggregation of platelets during hemostasis [26]. In addition to its prothrombotic properties, it stimulates the activation of new platelets [29]. Aspirin inhibition of COX-1 in platelets decreases the production of TxA_2_ [30] and, hence, the antithrombotic property of aspirin and its potential for cardiovascular protection [27]. Aspirin inhibits TxA_2_ synthesis, resulting in a decreased chance of thrombosis or thrombotic events.

## 2. NSAIDs Protect against Cancer: Proof of Principle

Cancer prevention entered a new era when it was recognized that subjects using NSAIDs had a lower incidence of colorectal cancer [31]. Over 30 epidemiological studies, collectively describing results on over one million subjects, have established NSAIDs as the prototypical chemopreventive agents against many forms of cancer. NSAIDs, both nonselective and COXIBs, have been associated with reduced cancer risk [32,33]. However, COXIBs are associated with a more significant reduction in cancer risk [32,33,34]. In a nested case–control study of the association between COXIBs and breast cancer risk, more extended and/or higher doses of celecoxib and rofecoxib were associated with significantly decreased risk [32]. In general, COXIBs have shown more substantial protective effects than nonselective NSAIDs against many cancers [32,33,35]. 

The use of NSAIDs is limited by their significant toxicity, which includes (i) gastrointestinal side effects, which range from dyspepsia to gastrointestinal bleeding, obstruction, and perforation; (ii) renal side effects; and (iii) a large number of additional side effects, some of which are serious, ranging from hypersensitivity reactions to the distinct salicylate intoxication [2]. Among patients using NSAIDs, up to 4% per year have serious gastrointestinal (GI) complications, with ~8000 deaths [36]. The gastric damage is caused through two distinct mechanisms: (1) direct epithelial damage as a result of their acidic properties; (2) breakdown of mucosal defense mechanisms (leukocyte adherence, decreases in blood flow, bicarbonate, and mucus secretions) due to a reduction in mucosal PG synthesis [37].

## 3. Molecular Targets of NSAIDs

In addition to COX, traditional NSAIDs and COXIBs can modulate many different signaling pathways, such as NF-κB, phosphodiesterases, NSAID-activated gene (NAG-1), peroxisome proliferator-activated receptors (PPAR), the Wnt pathway, cell kinetic effects, Akt pathway, and pro-resolving mediators. These are discussed below.

### 3.1. COX Cascade

**Figure 1:**
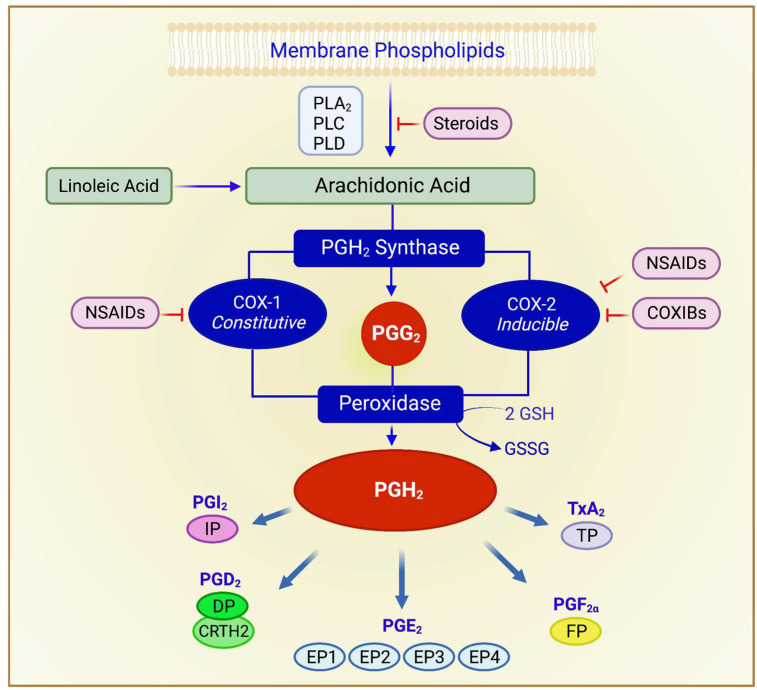
Overview of the cyclooxygenase pathway. Arachidonic acid, the substrate of the cyclooxygenase, (COX) biosynthetic pathways, is derived from diet or synthesized from linoleic acid and is released from membrane phospholipids through a series of reactions requiring phospholipases. The enzyme prostaglandin H2 synthase catalyzes the committed step. It exhibits two catalytic activities, cyclooxygenase and peroxidase. The enzyme expressing both activities is referred to as COX. There are two isoforms of PGH_2_ synthase, designated COX-1 and COX-2. The cyclooxygenase component of PGH_2_ synthase, produces the unstable intermediate PGG_2_, which is rapidly converted to PGH_2_ by the peroxidase activity of PGH_2_ synthase; this requires 2 equivalents of GSH. Specific isomerases convert PGH_2_ to various PGs and TxA_2_. Steroids have powerful anti-inflammatory properties because they inhibit phospholipases, thus limiting the bioavailability of arachidonic acid. Abbreviations: prostaglandin H_2_ synthase, PGH_2_ synthase; prostaglandin G_2_, PGG_2;_ prostaglandin H_2_, PGH_2_; phospholipases A_2_, C, and D, PLA_2_, PLC, PLD; glutathione, GSH; prostaglandins (respective receptors), PGE_2_ (EP1-4), PGF_2α_ (FP), PGD_2_ (DP, CRTH2); prostacyclin, PGI_2_ (IP); thromboxane A_2_, TxA_2_ (TP).

Conceptually, NSAIDs prevent cancer through their effects on the eicosanoid pathways. In the presence of molecular oxygen, the COX pathway produces PGH_2_, which is then converted to various PGs and TxA_2_ by specific isomerases [2,38] (Figure 1). Since human colon cancers have elevated PGE_2_ levels [39,40] and COX-2 is overexpressed in 45% of colon adenomas and 85% of colon carcinomas [41,42,43], COX-2 inhibition should, therefore, arrest carcinogenesis. Multiple in vitro and in vivo studies supported the notion that COXIBs have utility against many forms of cancers [44]. For example, in mice, deletion of COX-2 significantly decreased the number of intestinal tumors in *APC^D716^* mice [45]. In December 1999, the Food and Drug Administration approved the use of celecoxib in patients with familial adenomatous polyposis (FAP) [46].

The role of COX-2 as a central player in carcinogenesis was challenged very early on when it was shown that both sulindac sulfide and piroxicam induced apoptosis in COX-2-expressing HT-29 and the COX-2-deficient HCT-15 human colon cancer cells [47]; NS-398, a potent COXIB, had similar effects in HT-29 and S/KS (COX-2-negative) human colon cancer cell lines [48]. Furthermore, results were disappointing when COX-2 inhibitors were combined with chemotherapeutic agents to control cancer. For example, celecoxib combined with trastuzumab failed to affect patients with HER2/neu-overexpressing, trastuzumab-refractory metastatic breast cancer [49]. Preclinical studies had demonstrated a link between overexpression of HER-2/neu and COX-2 activity. Similarly, in a phase II study of metastatic colon cancer, rofecoxib combined with chemotherapy showed increased toxicity and no efficacy [50]. 

Recent data suggest that PGE_2_ produced by the tumor cells may contribute to a weakened immune system in cancer patients [26,51]. The secretion of PGE_2_ by tumor cells suppresses NF-κB, which plays a role in inducing the natural killer (NK) cells, thus preventing these immune cells from maturing and reducing their ability to damage cells. This leads to their ineffectiveness in combating infections and tumor growth and spread [51]. Furthermore, numerous studies have concluded that NSAIDs, such as indomethacin and modified aspirins, effectively reduce or mitigate immunosuppression in tumor-bearing mice [26]. 

#### COX-2 Inhibition

COXIBs inhibit COX-2 by a combination of hydrophobic interactions and hydrogen bonding to the enzyme’s active site [26]. COXIBs were developed to have similar efficacy as traditional NSAIDs, that is, to have anti-inflammatory, antipyretic, and analgesic properties without affecting the role of COX-1 in maintaining physiological functions [27,52]. They essentially offered the same benefits as nonselective NSAIDs, but without the consequential GI side effects [27,53]. However, in some patients that are at an increased risk of GI ulceration, the benefits of COXIBs over traditional NSAIDs are significantly reduced [54]. 

A number of studies [55,56,57,58], including some clinical trials, have shown that long-term use of COXIBs is associated with an increased risk of adverse myocardial events. For example, in the Vioxx Gastrointestinal Outcomes Research (VIGOR) studies comparing the upper GI safety of rofecoxib and naproxen in patients with rheumatoid arthritis, it was shown that the rate of serious GI events among those using rofecoxib was half compared to those using naproxen (2% vs. 4%). However, an increase by a factor of 4 in MI incidence was observed [59].

The Celecoxib Long-Term Arthritis Safety Study (CLASS) trial comparing the upper GI safety profile of celecoxib to that of ibuprofen or diclofenac in patients with osteoarthritis and rheumatoid arthritis showed that, within the first six months, there were fewer GI side effects and no increases in cardiovascular risk amongst the celecoxib treatment group [60]. However, after one year, celecoxib did not differ from the traditional NSAIDs on its GI effects [61]. Furthermore, a meta-analysis of 55 trials (99,087 patients) indicated a higher incidence of cardiovascular events in various COXIB users, including celecoxib [62]. Finally, the Adenomatous Polyp Prevention on Vioxx (APPROVe) study was launched to evaluate the efficacy of rofecoxib in patients with a previous history of colorectal adenomas and showed that 3.5% of rofecoxib recipients and 1.9% of placebo recipients suffered myocardial infarctions or strokes during the trial [63]. This prompted the termination of this and all related trials and voluntary withdrawal of rofecoxib. Reductions in prostacyclin (PGI_2_) due to COX-2 inhibition in the vasculature, thus reducing vasodilation with a consequent increase in thromboxane, were proposed as the underlying mechanism for this increased risk [64]. It has also been suggested that COX-2 inhibition in the kidneys leading to decreases in PGI_2_ could lead to increases in blood pressure and, hence, increases in myocardial infarctions and stroke [65]. It should also be noted that traditional NSAIDs can also precipitate untoward cardiovascular events under certain conditions [66].

## 4. NSAID Targets beyond COX

It is clear that NSAIDs prevent various human cancers and that they act on multiple molecular targets, of which COX-2 is only one. Table 1 represents the effects of NSAIDs on some of these non-COX targets that we have addressed in the following sections. 

### 4.1. NF-κB

The nuclear factor kappa-light-chain-enhancer of activated B cells (NF-κB) is a transcription factor relevant to the gene expression in inflammation and immune responses [139]. Many reports demonstrate that NF-κB is involved in the development of cancer. It is upregulated through chromosomal changes or constitutive activation in various hematological malignancies, such as lymphomas and solid tumors, including breast, ovarian, colon, pancreatic, and prostate cancer [140,141,142,143,144,145,146]. Activation of NF-κB in cancer increases cell proliferation and angiogenesis but suppresses apoptosis, all of which define the development of the tumor mass [67,147,148,149]. The NF-κB pathway is initiated by stimuli in the form of pathogens, infection, or wound and involves the subsequent activation of and communication between other inflammatory cytokines and receptors [150,151]. NF-κB is held inactive in the cytoplasm by IκB protein, which, upon phosphorylation, is proteasomally degraded, and NF-κB dissociates from this complex. Nuclear translocation of NF-κB brings it into contact with its binding site in target genes, allowing for transcription and expression of genes involved in inflammation and growth [38]. NF-κB can be activated by TNF-α, which leads to the expression of genes that code enhanced proinflammatory activity, thus linking TNF-α and NF-κB to inflammation and cancer [151,152,153]. Prolonged activation of NF-κB has been linked to carcinogenesis and tumor promotion [154].

Kopp and Ghosh were the first to demonstrate that aspirin inhibits the activation of NF-κB without interfering with the cell’s transcriptional machinery [68]. Prolonged treatment of colorectal cancer cells with aspirin decreases cytoplasmic IκBα, and thus increases translocation of NF-κB to the nucleus; such activation of the NF-κB pathway induced apoptosis in these cells [38,67,69,155]. This was also observed with diclofenac [70]. Sulindac decreases Iκκβ kinase activity, thus regulating the NF-κB pathway and reducing proliferation of colon cancer cells [71,72]. 

### 4.2. PDK-1/Akt Pathway

PDK-1 is a serine/threonine kinase that activates the phosphoinositide 3-kinase (PI3K) signal transduction pathway. When PI3K is activated and catalyzes phosphatidylinositol (4,5)-bisphosphate (PIP_2_) to phosphatidylinositol (3,4,5)-trisphosphate (PIP_3_) in the plasma membrane, PDK-1 is recruited to the membrane, where it binds to PIP_3_, and PI3K activates it. Then, PDK-1 phosphorylates and activates protein kinase B (Akt), which has downstream effects in cell survival and inhibition of apoptosis [156].

Akt is a serine/threonine kinase and, like many other protein kinases, is intimately involved in cell death and survival and has a pivotal role in many cell signaling pathways involved in angiogenesis, cell growth, division, apoptosis, and metabolism [157]. All available data show that Akt is hyperactive in cancer cells, and many types of human cancer are associated with the upregulation of Akt [158], for example, gastric carcinoma, glioblastomas, gliosarcomas, head and neck squamous cell carcinoma, pancreatic, ovarian, breast, and prostate cancers [159,160,161]. Inhibition of PI3K or targeting other molecules associated with this signaling network are bona fide targets in cancer treatment [162,163].

Celecoxib inhibits PDK-1 activity in colon cancer by blocking the phosphorylation of Akt at specific serine and threonine residues, thus promoting apoptosis [73]. In normal glomerular mesangial cells, celecoxib activated the PI3K signaling pathway by phosphorylating its downstream effector molecules [164]. However, in the prostate [74] and colorectal [73] cancer cell lines, celecoxib inhibited the PI3K pathway, causing apoptosis. In addition, naproxen treatment of urinary bladder cancer cells showed that its binding and inhibition of PI3K leads to the inhibition of Akt and further downstream effectors that play roles in apoptosis and cell survival [75]. Similar effects were seen with aspirin treatment of PIK3CA mutant colorectal cancer cells [165].

### 4.3. PPARs

The peroxisome proliferator-activated receptors (PPARs) are nuclear receptors that control the expression of genes involved in cell proliferation, growth, and inflammation. There are three isoforms of PPARs, α, β/δ, and γ; the a is involved in fatty acid catabolism, and thus is expressed in the related organs; β/δ is present in all cell types; and γ is involved in the differentiation of adipose cells [166,167]. PPARs heterodimerize with the retinoid X receptor (RTX). This complex interacts with specific response elements in the DNA of target genes to recruit cofactors, leading to increases in the transcription of these genes [166,167]. 

Although all PPAR forms are associated with cancer [168,169,170,171], PPARγ has anticarcinogenic properties, as it promotes apoptosis and restricts cell growth [172]. In a xenograft model using HCT116 colon cancer cells, the PPARγ agonist troglitazone inhibited the development of tumors [168]; also, feeding troglitazone to rats [173] and mice [174] decreased the incidence of colitis, which is a risk factor for colon cancer development in humans, and formation of aberrant crypt foci, an early step in the development of colon carcinoma [172]. However, using the APC*^Min^* mouse model, treatment with troglitazone increased the number of tumors [175,176]. The conundrum of PPARγ in colon carcinogenesis suggests that early treatment with the PPARγ agonists before the first step of carcinogenesis occurs may prevent tumor formation, but that activation of PPARγ after tumor initiation, as is the case in the APC*^Min^* mice, may promote cancer [172,177]. High-fat diets may also lead to colon cancer formation potentially due to PPARγ activation [177].

The role of PPARβ/δ in cancer is controversial, with some studies suggesting that it promotes carcinogenesis, while others suggest that it may inhibit it [178]. PPARβ/δ enhances colon cancer formation by inhibiting differentiation, promoting cell movement, and causing apoptotic resistance [179]. PPARβ/δ, which is highly expressed in colon cancer cells, was suppressed by the *APC* gene via the β-catenin/Tcf-4 response elements in its promoter region [77], although this mechanism is debatable, as disruption of *APC* in the mouse colon does not increase PPARβ/δ expression [180]. Furthermore, PPARβ/δ-null *Min* mice showed increased intestinal tumorigenesis [180]. 

As PPARs are receptors for products of arachidonic and linoleic acids, they are affected by NSAID exposure. NSAIDs inhibit PPAR-δ expression and activity, thus reducing colorectal cancer promotion; this is mediated by the NSAID-induced reductions in 15-LOX-1 expression [181]. 15-LOX-1 and 15-LOX-2 are enzymes in the lipoxygenase (LOX) pathway and have anticarcinogenic effects (reviewed in [2,38]) (Table 1).

The preferred substrate for 15-LOX-1 is linoleic acid, and for 15-LOX-2 is arachidonic acid [182]. 15-LOX-1 is the main enzyme for metabolizing linoleic acid into 13-*S*-HODE [183] and is the only 15-LOX isozyme found in the human colon epithelium [184]. 13-*S*-HODE is linked to cellular differentiation and apoptosis, 15-LOX-1 expression levels are reduced in human colorectal cancers, and NSAIDs induce its expression, which is COX-2-independent [185], as reviewed in [38].

Aspirin, ibuprofen, and indomethacin are ligands for PPARγ, thus activating the receptor and inducing adipocyte cell differentiation and increasing peroxisomes’ activity in hepatocytes [76]. Therefore, there is a potential role for NSAIDs via activation to induce the anticarcinogenic properties of PPARγ. 

For PPARβ/δ, its tumorigenic properties may be inhibited by NSAIDs. Sulindac and indomethacin impaired the ability of PPARβ/δ to bind to its DNA recognition sequences, resulting in decreased activity, similar to that observed in the APC model [77]. However, sulindac also induced apoptosis in both wild-type and PPARβ/δ-null HCT116 colon cancer cell lines [78], suggesting that NSAID-induced inhibition of PPAR is not the main mediator of sulindac-induced apoptosis [186]. Furthermore, NS398 in combination with rosiglitazone caused synergistic apoptosis and growth inhibition in SW1990 human pancreatic cancer cells [79]. Celecoxib in combination with DHA had greater growth inhibition and apoptosis induced in prostate cancer cells (LNCaP, PC-3, and DU145) treated with lower doses of DHA/celecoxib combination therapy than in cells treated with higher doses of the individual agents [80]. 

### 4.4. Mitogen-Activated Protein Kinases (MAPKs)

The MAPKs are a family of serine/threonine kinases that transduce signals from the cell membrane to the nucleus in response to various stimuli, modulating gene transcription and leading to biological responses (reviewed in [187,188]). The MAPKs in mammals include p38, c-Jun N-terminal kinase (JNK), and extracellular-signal-regulated kinase (ERK1/2) [188,189,190], which are important in inflammation [191,192], arthritis [191], and cancer [190,193,194]. Mitogens and differentiation-related signals activate ERK signaling, while p38 and JNK MAPKs are activated by inflammation and stress stimuli [187]. The ERK signaling is constitutively active in cancer, promoting progression through the cell cycle and growth [86,195]. JNK is activated by dual phosphorylation at tyrosine and threonine residues; it phosphorylates serine residues on c-Jun, an oncoprotein and transcription factor [190,196]. P38 and JNK activity induce apoptosis of lung tumor cells [197]. JNK and p38 MAPKs were highly expressed in the stroma cells of human colon adenomas [198]. Increased JNK activity was observed in osteosarcoma cells with high c-Jun and phosphorylated (activated) c-Jun present in higher-grade tumors, suggesting the role of the JNK signaling pathway in tumor progression [196].

In many human cancers, Ras is frequently mutated [199,200]; this includes cancers of the colon [201,202], lung [203], and pancreas [204]. Ras mutations were found in over one third of human colorectal cancers [201]. K-Ras and N-Ras are members of the small G-proteins superfamily and act as switches, regulated by GDP and GTP, to communicate signals from the extracellular environment to intracellular signaling and response pathways. When mutated, these proteins remain in the GTP-bound state and are activated [205]. Mutations in the Ras oncogene are associated with tumor progression from adenoma to carcinoma. They were found in 58% of colorectal adenomas bigger than 1 cm, compared to 9% of those smaller than 1 cm, and in 47% of colorectal adenocarcinomas [206]. K-Ras mutations, which frequently occur in colon cancers, play an initiating role in neoplasia [201,207]. It activated MEK/ERK signaling and increased cell proliferation in the proximal colon [207]. Human colon cancer cells with the K-Ras mutation exhibited hyperproliferation due to hyperactivation of the MEK/ERK pathway, while N-Ras mutation conferred apoptotic resistance to colon cancer cells [200].

NSAIDs induce p38 [208] and JNK activation [209], thus providing another target for their chemopreventive properties. NSAIDs have been shown to mediate the MAPK signaling pathway in numerous cancers, including that of gastric [81], renal [82], liver [87], colorectal [83,84,85,86], and head and neck cancers [88]. Indomethacin and NS-398 reduced cell growth and proliferation and induced apoptosis in MKN28 human gastric cancer cell lines by inhibiting the ERK2 MAPK signaling pathway [81]. When indomethacin treated 786-O renal carcinoma cells, p38 and JNK activity and apoptosis increased [82]. Celecoxib lowered ERK signaling in mice models of ulcerative-colitis-associated colon cancer [86]. In head and neck squamous cell carcinoma, celecoxib upregulated ERK and/or p38 signaling and inhibited cell growth; when these ERK and/or p38 signaling pathways were blocked, the celecoxib-induced cell growth inhibition was significantly reversed [88]. In mice with liver cancer, celecoxib inhibited ERK activity and increased activation of p38 and JNK signaling, thus blocking cancer growth and inducing apoptosis of cancer [87]. When the COXIB NS-398 was used to treat HT29 colorectal cancer cells, there was sustained activation of ERK by MEK, followed by lower cell yield and increased apoptosis, suggesting a proapoptotic role by NSAID-induced ERK activity [84]. These HT29 cells also exhibited sustained activation of the MAPKs and induced apoptosis in response to indomethacin [83]. Sulindac sulfide and NS-398 treatment of HCT-15 and HCA-7 colon cancer cells also showed time- and dosage-dependent activation of the MEK/ERK and p38 MAPKs. However, sulindac sulfide exhibited greater ERK activation than NS-398 [85].

### 4.5. Wnt/β-Catenin Pathway

The Wnt/β-catenin pathway is a characteristic signaling cascade of tumors and cancers [38,210,211]. In normal function, Wnt/β-catenin signaling is critical for developing, differentiating, and growing immune and stem cells [212,213]. The involvement of the Wnt/β-catenin pathway in response to pathogens is reviewed in [213], and, in cancers, in [214,215]. Dysregulated Wnt/β-catenin signaling poses disastrous effects on the immune system, leading to incidences of uncontrolled inflammation and poor immune defense [212,216]. It dampens leukocyte infiltration to the inflamed site, and the resulting growth and invasion of the tumors have been well studied (reviewed in [212]). In addition, wnt/β-catenin signaling has been shown to promote and counter inflammatory activity in relation to its communication with NF-κB [217]. 

In carcinogenesis, the β-catenin pathway in dendritic cells, which are important for achieving adaptive immunity and blocking autoimmune attacks [218], is activated by tumors that allow them to avoid surveillance by the body’s immune system and continue to grow and spread [216]. The Wnt/β-catenin pathway is implicated in colorectal [219,220,221], breast [222,223,224], and hematological [89,225,226,227] cancers, as well as in melanoma [228,229,230,231].

NSAIDs target the Wnt/β-catenin signaling pathway in colorectal [38,232], gastric [233], leukemia [89], lung [90], glioblastoma [93], and breast [90,94] cancer cell lines. Treatment of chronic myeloid leukemia stem cells with indomethacin resulted in lower levels of β-catenin and decreases in leukemia stem cells, thus delaying oncogenesis [89]. Sulindac treatment of colon cancer cells led to downregulation and degradation of β-catenin in a dose- and time-dependent manner, inhibiting tumor growth [91]. Moreover, sulindac treatment of the colorectal cancer cell lines DLD1 and SW480 resulted in lower β-catenin expression; furthermore, sulindac prevented nuclear translocation and the transcriptional activity of β-catenin [92]. Treatment of human glioblastoma cells with diclofenac and celecoxib resulted in decreased cell proliferation and migration, along with reduced β-catenin expression and suppression of the Wnt/β-catenin signaling pathway [93]. 

### 4.6. Phosphodiesterases (PDEs)

Phosphodiesterases (PDEs) are enzymes that cleave phosphodiester bonds and catalyze the hydrolysis of cyclic nucleotides, such as cAMP and cGMP [234]. Phosphodiesterases are implicated in cancer because apoptosis and inhibition of cancer cell growth and migration are promoted when cAMP levels are high. Cancer cell invasion and migration are decreased by higher cAMP levels [235]. Thus, PDE inhibitors may be potential anticancer treatments [234,236]. They promote the increase in intracellular cAMP levels and induce/effect apoptosis at levels similar to chemotherapeutic drugs cisplatin and gemcitabine [236]. Sildenafil and vardenafil, which selectively inhibit PDE5 and PDE6, respectively, result in higher cGMP levels and effectively induce apoptosis in B-chronic lymphocytic leukemia cells in a caspase-dependent manner, with vardenafil being three times as effective as sildenafil [237]. 

Sulindac and its derivatives highlight the involvement of the cGMP/PDE/PKG signaling pathway in cancer. PDE5 promotes tumorigenesis and is overexpressed in tumors of the colon, lung, and breast [95,238,239,240]. Sulindac sulfide inhibited PDE5, resulting in higher intracellular cGMP levels, thus activating protein kinase G (PKG) [95,96]. PKG goes on to phosphorylate and activate β-catenin, an oncoprotein, thus inducing the degradation of the non-phosphorylated and oncogenic β-catenin pool and the subsequent inhibition of Wnt/β-catenin signaling and transcriptional activity and, hence, cell proliferation [96,97,98,239,240].

### 4.7. NSAIDs and the mTOR Pathway

Mammalian target of rapamycin (mTOR) is a serine/threonine kinase that is part of the PI3K family and is the core catalytic component of the mTOR complexes 1 (mTORC1) and 2 (mTORC2) [241]. mTOR is a downstream effector of the Akt signaling pathway and plays key roles in cellular growth, metabolism, and proliferation [242]. Both the MAPK and PI3K/Akt pathways result in the activation of mTORC1. mTORC2 increases phosphorylation and activation of Akt, thus promoting continued activation of mTORC1. The mTOR pathway, which regulates tumor metabolism and promotes growth and metastasis [243], is activated in many cancers, including that of breast [244,245], prostate [246], hepatocellular [247], pancreatic, renal, and melanoma cancers [248]. Aspirin inhibits mTOR signaling, inducing autophagy in HCT116, SW480, and RKO human colon cancer cell lines [99]. Celecoxib sensitized non-small cell lung cancer cells to radiation therapy, in part due to decreased mTOR expression [100].

### 4.8. Autophagy

Autophagy is part of the programed cell death process. In autophagy, the material is endocytosed by autophagosomes, which then fuse to lysosomes so that hydrolytic enzymes break down the contents for them to be recycled by the cell (reviewed in [249,250]). Normally, autophagy is used to clear debris and damaged organelles. However, increasing autophagy activity would induce autophagy cell death, an irreversible and nonapoptotic mechanism of programed cell death induced by some anticancer therapies [251,252]. Cytoprotective autophagy protects cancer cells in nutrient-poor or treatment-rich environments [249,253]. Induced autophagic death improves tumor outcomes, as it acts as a self-destruction mechanism; conversely, inhibiting cytoprotective autophagy can increase treatment efficacy [249,254]. 

Autophagic activity is lower in cancer than in normal cells [249]. However, this activity increases when cancer cells are treated with chemotherapeutic drugs [251,252]. In addition, autophagy may contribute to tumor cell survival when in dormancy [255].

Activation of mTORC1 inhibits autophagy. Under hypoxia and in the tumor microenvironment, the PI3K/Akt/mTOR signaling pathway is activated, inhibiting autophagy [256]. Thus, mTOR inhibitors induce autophagy [257]. NSAIDs can induce autophagic cell death via many targets, including the PI3K/Akt, mTOR, and MAPK pathways. For example, celecoxib suppressed cytoprotective autophagy while inducing apoptosis and necrosis in HL-60 acute leukemia cells [101] and HCT116, SW480, and HT-29 colorectal cancer cells [102], as well as NTUB 1 and T24 urothelial carcinoma [103] cells. Another COXIB, meloxicam, was shown to promote autophagic activity in HepG2, Bel-7402, Huh-7, SMMC-7721, and SMMC-7402 hepatocellular carcinoma cells [104,105]. Aspirin treatment enhances autophagy in human cancer cells in the short term, 12 h, but inhibits autophagic activity and induces apoptosis in the long term, 48 h, in cancers [106]. Autophagy is also induced by aspirin in HCT116, SW480, and RKO colorectal cancer cells [99].

### 4.9. Cell Kinetics

The first report suggesting that NSAIDs inhibit cancer cell growth by a COX-independent mechanism was published in 1996 [47]. Evaluating the effects of sulindac and piroxicam on cell kinetic parameters of two human colon cancer cell lines, HT-29, which expresses both COX-1 and COX-2 enzymes, and HCT-15, which is COX-null, it was shown that cell proliferation, cell cycle arrest, and apoptosis were independent of their inhibitory effect on PG synthesis [47]. 

Effects of celecoxib and SC560, a selective COX-1 inhibitor, were similar in other colon cancer lines that have different levels of COX-2 expression. Both NSAIDs resulted in a G_0_/G_1_ cell cycle arrest and lower survival rates regardless of COX-2 expression levels [107].

In urinary bladder cancer cells, naproxen induced G_1_ cell cycle arrest by inhibiting cdk4, cyclin D1, and increasing p21 expression, leading to higher apoptosis by increasing cleavage of caspases 3 and 7 [75]. 

In breast and ovarian cancer cells, sulindac sulfide increased p21 expression and cell cycle arrest, while decreasing cyclin D1 expression [108].

### 4.10. Cytochrome C Release

Apoptosis is mediated by cytochrome c, Bcl-2 family proteins, and caspases. Apoptosis initiation requires proapoptotic Bcl-2, multidomain Bax and Bak, and the BH-3-only proteins (Bid, Bad, Noxa, Puma, Bim), which detect death or damage cues [258]. These BH-3-only proteins induce the translocation of Bax and Bak to the mitochondrial outer membrane, where they form a channel through which cytochrome c leaks out from the mitochondria into the cytoplasm [258,259,260]. The cytochrome c leaked from the mitochondria binds to Apaf-1 and procaspase-9 to form the apoptosome, resulting in the activation of caspases that cleave cellular proteins to mediate apoptosis [260,261,262]. 

Indomethacin induced cytochrome c release in Caco-2 cells, which are of human intestinal epithelial origin, by increasing Ca^2+^ concentrations in the mitochondrial matrix [109]. Meanwhile, in the same cell line, celecoxib increased caspase-9 and caspase-3 activity [111]. In three colon cancer cell lines (HCT-15, SW620, SW480), NS398 (a COX-2 selective inhibitor) induced apoptosis via mitochondrial cytochrome c release [113]. Aspirin also induced cytochrome-c-dependent apoptosis in HeLa cervical cancer cells [114]. In MGC803 gastric cancer cells, celecoxib induced apoptosis through cytochrome c release and zymogen cleavage and activation of caspase-9 [112]. In esophageal cancer cell lines, indomethacin treatment resulted in higher expression of proapoptotic Bax protein, cytochrome c release, and caspase-9 activation [110].

### 4.11. NSAID-Activated Gene (NAG-1) 

NAG-1 is a divergent and unique member of the TGF-β superfamily of proteins, which are important in growth and development. NAG-1 has antitumorigenic properties and promotes apoptosis [115,263,264]. Its expression is upregulated by NSAIDs such as aspirin and ibuprofen in various cancers, such as colon [115,116], breast [119], pancreas [122], prostate [123,265], glioblastoma [117], ovarian [108], and gastric cancers [118,120]. NAG-1 expression is mediated by several tumor suppressor pathways, including p53, GSK-3β, and EGR-1 [263]. NAG-1 induction following NSAID treatment is also mediated by the p38 MAPK pathway [123]. NSAID treatment of HCT-116 colon cancer cells that are COX-null resulted in higher NAG-1 expression and higher levels of basal apoptosis [115,116], thus exhibiting COX-independent effects. Furthermore, NAG-1-transfected cells exhibited increased basal apoptosis and increased response to NSAIDs. When tumors derived from NAG-1-transfected HCT-116 cells were transplanted in athymic nude mice, they showed reduced tumorigenicity [116]. 

NAG-1 expression was upregulated, while COX-2 was inhibited, by celecoxib administration to APC^Min/+^/NAG-1^Tg/Lox^ (human NAG-1) mice [121]. In yet another study, sulindac sulfide was identified as the most potent inducer of NAG-1 and it was concluded that NAG-1 is a mediator in sulindac sulfide inhibition of ovarian cancer growth [108]. NSAIDs that promote apoptosis can also increase NAG-1 expression and strongly inhibit COX enzymes, demonstrating that NAG-1 may play a role in these proapoptotic properties [38]. NAG-1 expression is higher in many cancers, and abnormal increases correlate with cancer severity and survival. NAG-1 has been shown to prevent tumor growth and development in prostate [123] and colorectal cancers [115,116]. It was also found that NAG-1 induction by ibuprofen and flurbiprofen inhibited the migration of prostate PC-3 cancer cells [123]. In addition, NSAIDs have been linked to metastasis suppression in various cancers, including the prostate. Thus, a potential mechanism of NSAID-mediated metastasis suppression may be through the induction of NAG-1 [123]. 

### 4.12. Ca^2+^ Mobilization

Ca^2+^, a ubiquitous secondary messenger, regulates various cellular processes, such as survival, growth, differentiation, and death, by activating or inhibiting cellular signaling pathways and Ca^2+^-regulated proteins. Three main classes of membrane-bound proteins that have diverse tissue expression are directly involved in Ca^2+^ regulation; these are (i) exchangers, (ii) channels, and (iii) ATPases (pumps) [266,267]. Dysregulation of any of these exchangers, channels, and pumps or Ca^2+^-binding proteins (such as calmodulin, calnexin, calpains, and others) are involved in many cancers, including those of colon [268], breast [269], pancreas [270], prostate [271], skin [272], leukemia [273], lung [269], and others. 

Celecoxib treatment of prostate cancer PC-3 cell line blocked Ca^2+^ reuptake via inhibition of the Ca^2+^ channels on the endoplasmic reticulum, resulting in Ca^2+^ intake into the cell and release of Ca^2+^ by the ER [125]. This increases Ca^2+^ concentration and triggers the ER-associated stress response, resulting in apoptosis. This behavior is unique only to celecoxib but is observed in various cancer cell types, including Jurkat T cells and NIH 3T3 fibroblasts [125]. This antitumor effect was also seen with 2,5-dimethyl-celecoxib (DMC), an analog that lacks COX-2-selective specificity [126]. When comparing celecoxib with DMC on Ca^2+^ mobilization, celecoxib inhibited the ER Ca^2+^ ATPases stronger than DMC, though DMC showed a greater potency when measuring Ca^2+^ transport [127]. Indomethacin treatment of HT29 colon cancer cells inhibited Ca^2+^ entry into the cell in a dose-dependent manner that decreased its intracellular concentration, thus blocking cell migration and preventing tumor metastasis [124]. When human intestinal epithelial Caco-2 cells were studied, there was increased Ca^2+^ release from the ER through the IP3 and ryanodine receptors, resulting in cytotoxicity and apoptotic events [109]. 

### 4.13. Inhibition of Angiogenesis

Angiogenesis is the process by which new blood vessels form, allowing the delivery of oxygen and nutrients to the body’s tissues. It also plays a vital role in cancer formation, since tumors also need a blood supply to thrive and grow (reviewed in [274,275]). Angiogenesis inhibitors have been used to treat cancer. They lead to apoptosis with no associated changes in cell proliferation, suggesting that apoptosis results from the lack of growth and survival factors secreted by the endothelial cells in the tumor microenvironment [276,277]. Treatment of tumor metastases T241 fibrosarcoma and Lewis lung carcinoma cell lines with an angiogenesis inhibitor TNP-470 was found to prolong tumor dormancy by increasing the apoptosis rate without affecting the proliferation of the tumor cells [276]. In human adenocarcinomas, endogenous inhibitors of angiogenesis, such as thrombospondin-1, are downregulated, while proangiogenic vascular endothelial growth factor (VEGF) is upregulated [277,278,279]. The increased angiogenic activity allows micrometastases to leave this dormant stage and spread to other areas of the body [276]. 

As angiogenesis involves both COX isoforms, NSAIDs play a role in inhibiting angiogenesis. Mechanisms of NSAID-induced blockage of angiogenesis include changes in the profiles of angiogenic growth/survival and inhibitory factors, VEGF expression, inhibition of the MAPK (ERK2) signaling pathway, and obstruction of the localization of ERK to the nucleus [280].

NSAIDs have been shown to inhibit angiogenesis in various cancer types, including gastric [130], breast [128,129], and colorectal cancers [131]. For example, low-dose therapeutic levels of ibuprofen reduced tumor growth and metastases of colorectal cancer human HT-29 and mice MC-26 cell lines by blocking angiogenesis. Furthermore, they did so without a higher risk of GI toxicity [131]. Ibuprofen also exhibited similar results when studied in gastric adenocarcinoma [130]. In addition, aspirin-induced blockage of angiogenesis and tumor metastasis were observed with the estrogen-receptor-negative human breast cancer MDA-MB-231 and MDA-MB-435 cell lines and the murine melanoma B16F10 cell line [128].

### 4.14. Carbonic Anhydrase (CA) Inhibition

Carbonic anhydrases (CAs), of which there are 15 isoforms, are enzymes that catalyze the reversible conversion of carbon dioxide (CO_2_) to bicarbonate ion and proton and are present in all body cells [281]. Of the 15 isoforms, CA IX and XII isozymes are tumor-associated because they are induced under hypoxic conditions [282,283]. In addition, the presence of CAs in the tumor microenvironment allows for pH regulation in response to changes induced by hypoxia, thus aiding tumor cell invasion and adhesion and, thus, metastasis [284].

Indomethacin increased the activity of CA I and II isozymes from human and bovine erythrocytes, respectively, in a dose-dependent noncompetitive manner [132]. It was suggested that CA and COX activity are inversely related, in that pH changes due to CA being activated result in inhibition of COX activity [132]. Celecoxib inhibited human CA II of the renal tubule cells with comparable affinities as the classical CA inhibitor (CAI), dichlorophenamide (DCP) [133,134], and both celecoxib and valdecoxib potently inhibited CA IX with effectiveness that was stronger than the best CAIs, acetazolamide and methazolamide [133]. This effect displayed by celecoxib and valdecoxib was thought to occur because they possess the sulfonimide moiety present in CAIs [133,134].

CA IX and XII isozymes were overexpressed in tumors and are markers for hypoxia in the tumor microenvironment [285]. They were observed in various cancer types, including cervical [286], breast [287,288], ovarian [285], pancreas [289], and brain cancers [290]. In a study on breast cancer MDA-MB-231 and ZR-75-1 cell lines, all metastatic lymph nodes studied expressed either CA IX or CA XII, or both isozymes [287]. A retrospective study on CA IX expression in patients who had invasive breast cancer found that CA IX expression was a significant predictor of survival. Patients with CA-IX-positive tumors had 2.5 times the risk of death as patients with CA-IX-negative tumors [288]. The poor prognosis of CA IX was also observed in oligodendroglial brain tumors [290].

## 5. Specialized Pro-Resolving Mediators (SPMs)

**Figure 2:**
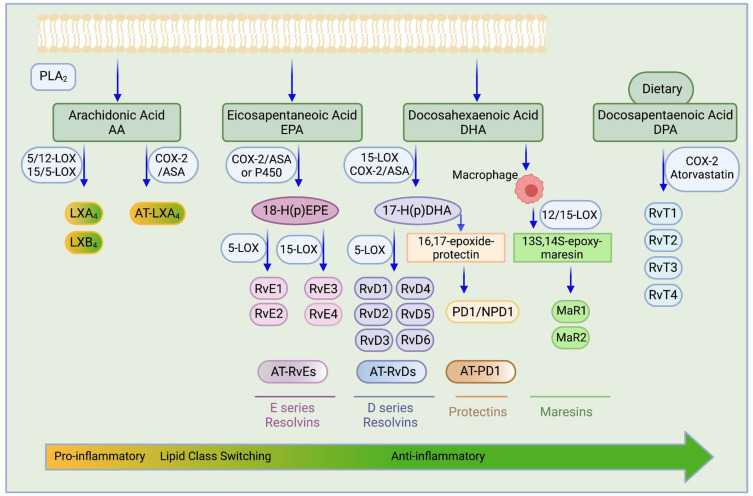
Production of special pro-resolving mediators (SPMs). Biosynthesis of lipoxins and aspirin-triggered (AT) lipoxins from arachidonic acid (AA); biosynthesis of resolvins and aspirin-triggered (AT) resolvins, protectins and aspirin-triggered (AT) protectins, and maresins from eicosapentaenoic acid (EPA) and docosahexaenoic acid (DHA); biosynthesis of the T series resolvins from docosapentaenoic acid (DPA). Abbreviations: RvE1, resolvin E1; RvE2, resolvin E2; RvE3, resolvin E3; RvE4, resolvin E4; AT-RvEs, aspirin-triggered E-series resolvins; LXA4, lipoxin A4; LXB4, lipoxin B4; AT-LXA4, aspirin-triggered lipoxin A4; 15-LOX, 15-lipoxygenase; 5-LOX, 5-lipoxygenase; 12-LOX, 12-lipoxygenase; COX2/ASA, aspirin-acetylated cyclooxygenase-2; P450, cytochrome P450; RvD1, resolvin D1; RvD2, resolvin D2; RvD3, resolvin D3; RvD4, resolvin D4; RvD5, resolvin D5; RvD6, resolvin D6; AT-RvDs, aspirin-triggered D-series resolvins; PD1/NPD1, protectin D1/neuroprotection D1; AT-PD1, aspirin-triggered protectin D1; MaR1, maresin 1; MaR2, maresin 2; RvT1, resolvin T1; RvT2, resolvin T2; RvT3, resolvin T3; RvT4, resolvin T4.

The resolution phase of acute inflammation is an active process whose purpose is to clear polymorphonuclear leukocytes (PMNs) and cellular debris from the injury site [291]. This phase engages in cleaning the inflammation site and restoring homeostasis [291,292,293]. This includes removing the cause for infection or injury, deactivating proinflammatory signals and their pathways, and the efferocytosis of apoptotic PMNs via phagocytosis by monocyte-derived macrophages [291,294]. Not only that, but lipid mediators also involved in the resolution phase modulate chemokine gradients such that the acute inflammation response is concluded and homeostasis is achieved. These endogenous anti-inflammatory bioactive lipid mediators are called specialized pro-resolving mediators (SPMs), biosynthesized locally and in a time-dependent manner to promote the resolution of inflammation and subsequent tissue healing [291,292,293,295]. SPMs are synthesized from the essential fatty acids and include the lipoxins (LXs) [296], resolvins (Rvs) [295], maresins (MaRs) [297], and protectins (PDs) [297]. Of these, LXs are AA-derived, while Rvs, MaRs, and PDs are derived from omega-3 PUFAs, EPA, and DHA [298] (Figure 2).

As omega-3 fatty acids, EPA and DHA decrease cancer risk [299,300,301,302] by suppressing the biosynthesis of eicosanoids from AA. High dietary consumption of DHA and EPA leads to their greater incorporation into the phospholipid bilayer of the cell membrane, competing for position against AA. Decreased availability of AA precursors allows for the production of EPA-derived prostanoids and leukotrienes. Not only that, the greater presence of omega-3 fatty acids reduces the conversion of linoleic acid (LA) to AA and, consequently, the generation of AA-derived eicosanoids. The lower AA level dampens COX-2 activity, which results in reduced formation of proinflammatory eicosanoids. Since the LOX pathway prefers EPA as a substrate, there is an increased production of EPA-derived LOX products [292]. The inhibited formation of AA-derived eicosanoids by omega-3 fatty acids and their derived eicosanoids suggests their anti-inflammatory and anticarcinogenic potential. 

Current cancer therapy aims to suppress the proinflammatory mechanisms and mediators; a novel approach would be promoting and enhancing the resolution phase of inflammation [303]. In cancer treatment, the focus has been on killing tumor cells to curb tumor growth and/or metastasis; this results in the buildup of dead cells or cellular debris that must then be cleared from the sites of inflammation [304]. Engaging the resolution processes would be necessary to remove apoptotic tumor cells and immune system components. In addition, the activity of resolution-promoting mediators may stimulate further healing and the dissipation of the accompanying surge of proinflammatory cytokines, also known as cytokine storm, present during uncontrolled inflammation [305,306]. NSAIDs, such as aspirin, reduce inflammation and prevent cancer. Therefore, it has been suggested that particular focus be placed on the potential of aspirin in triggering the production of these resolution-promoting mediators (resolvins, lipoxins, protectins, and maresins), which show promise in inhibiting cancer growth and spread (reviewed in [303]). 

### 5.1. Lipid Class Switching

PGs and leukotrienes (LTs) initiate the inflammatory response by attracting neutrophils to the site of injury or infection. After some time, the lipid profile begins to change, mediated by the expression/activation of the biosynthetic enzymes necessary for the generation LXs, Rvs, PDs, and MaRs [307]. PGE2 and PGI2 play crucial roles in the switch of these lipid profiles. Intercellular interactions between the lipoxygenase enzymes in PMNs recruited to the injury site and in inflamed tissue cells result in the switch in the synthesis of LTs to the generation of pro-resolving mediators, thereby enhancing resolution mechanisms [308]. The class-switching of eicosanoids involved in inflammation allows for the effective initiation, progression, and termination of the inflammatory response (Figure 2). 

#### 5.1.1. Lipoxins

Engaged extensively in the inflammatory response, LXs, along with their C15 epimers, aspirin-triggered lipoxins (AT-Ls), play crucial roles in the resolution phase (reviewed in [291,309,310]). LXs are produced through transcellular biosynthesis involving interactions between stimulated neutrophils and different cell types [309]. The enzyme 15-LOX-2 acts on AA to form 15-*S*-HETE, which binds as a substrate to 5-LOX in leukocytes to form the lipoxins LXA_4_ and LXB_4_. Alternatively, lipoxins can be synthesized by transforming AA to leukotriene LTA_4_ by 5-LOX; LTA_4_ then binds to platelet 12-LOX to generate lipoxins [307]. In this pathway, the initial steps are the same for the generation of leukotrienes. Thus, proinflammatory activity may be mediated through mechanisms that convert LTA_4_ (proinflammatory) to LX (anti-inflammatory), in addition to the direct anti-inflammatory effects of LXs. The third biochemical pathway leads to the formation of epi-lipoxins, or aspirin-triggered lipoxins (AT-Ls), which occur during cell–cell interactions, such as between endothelial cells and PMNs. Aspirin-acetylated COX-2 binds AA and mirrors 15-LOX-2 activity by oxygenating AA to form 15-*R*-HETE. Further oxygenation by 5-LOX generates the epimeric forms of lipoxins or ATLs [311]. 

The anti-inflammatory actions of LX/ATL include sending a stop signal to end the chemotaxis of PMNs to the inflamed tissue, inhibiting proinflammatory cell signaling to prevent tissue damage, and co-ordinating cellular interactions and responses to promote resolution and wound healing [312]. In addition, LXA_4_ increases the enlistment of macrophages to the site of inflammation and decreases the production of cytokines to mediate the inflammatory response, such as to clear away bacteria and signaling pathways. In this manner, LX/ATL closely mirrors the functions of resolvins [291].

Lipoxins may be useful targets for the induction of resolution and/or chemoprevention during carcinogenesis. Cancer cells, not just normal cells, can also synthesize lipoxins when treated with aspirin. These lipoxins, as autocoids, can exert their antitumor effects on the cancer cells that produce them. For example, co-stimulation of aspirin-treated A549 human lung adenocarcinoma epithelial cells with PMNs produced LXA_4_, LXB_4_, and their aspirin-triggered epimers; these LXs inhibited cell proliferation, with AT-LXB_4_ showing more potency than LXB_4_. However, cell growth inhibition of A549 cells by LXA_4_ and AT-LXA_4_ were affected to the same extent [135].

#### 5.1.2. Resolvins

Resolvins are oxygenated metabolites formed from the metabolism of omega-3 fatty acids. EPA produces the E-series resolvins (RvEs), and DHA forms the D-series resolvins, maresins, and protectins, while DPA gives rise to the T-series resolvins (Figure 2). 

RvEs are made during interactions between endothelial cells and leukocytes. RvE1 and RvE2 are made by two pathways: (i) cytochrome p450 and (ii) aspirin-acetylated COX-2. Aspirin acetylation of the COX-2 enzyme inhibits its ability to produce PGs but does not affect its peroxidation function, thus allowing the formation of anti-inflammatory mediators [313]. Occurring in the epithelial and endothelial tissues, cytochrome P450 [314] or aspirin-triggered COX-2 [313] acts upon EPA to form the intermediate 18-hydroxyeicosapentaenoic acid (18-HEPE). 18-HEPE interacts with the 5-LOX of the PMN leukocytes to produce RvE1 and RvE2 [315]. RvE1 may also be synthesized from the catalysis of 18-HEPE by the enzyme LTA_4_ hydroxylase, which also plays a key role in the formation of leukotrienes [313]. When human upregulated COX-2 endothelial cells were treated with indomethacin, acetaminophen, and aspirin, EPA formed 15-*R*-HEPE and 18-*R*-HEPE, which are intermediates for resolvins and are themselves bioactive [316]. 

In vascular endothelial cells, DHA reacts with 15-LOX and later 5-LOX to generate the 17-*S*-D-series resolvins (RVD1-6). In the presence of aspirin, DHA is catalyzed by aspirin-acetylated COX-2, followed by 5-LOX to produce the 17-*R*-resolvins, aspirin-triggered D-series resolvins (AT-RvDs) [317,318]. Resolvin D1 (RvD1) is associated with decreased leukocyte infiltration into inflamed tissue and augments the phagocytosis and removal of macrophages in the inflammatory response [293]. RvD1 and AT-RvD1 regulate leukocyte infiltration in a dose-dependent manner as oxidoreductases convert 17S configuration to 17-oxo-RvD1, its inactive form, which differs from the AT-RvD1, which is resistant to rapid inactivation [318]. Resolvin D2 (RvD2) has been shown to reduce the production of cytokines and the recruitment of immune cells to the site of inflammation. It possesses complex roles in the inflammatory response, influencing leukocyte transmission from plasma to inflamed tissue and the levels of cytokine and eicosanoid products [291]. 

The T-series resolvins (RvTs), also called 13-series resolvins, are derived from n-3 docosapentaenoic acid (DPA). RvTs are synthesized via neutrophil–endothelial cell interactions in which COX-2 converts DPA to 13-*R*-hydroperoxyDPA (13-R-HpDPA), which is hydrolyzed to 13-hydroxyDPA (13-*R*-HDPA) and then converted to resolvins in neutrophils; its synthesis is increased by atorvastatin, which results in *S*-nitrosylation of COX-2 [319]. RvTs (1-4) inhibit macrophage-mediated activation of inflammasomes and activity of proinflammatory cytokine IL-1b. Like other SPMs, DPA-derived resolvins stimulate pro-resolving activities, while also exhibiting anti-inflammatory properties, including enhancing phagocytosis of cell debris and infectious agents [319]. 

Aspirin-triggered resolvins exhibit anticarcinogenic and pro-resolving properties without immunosuppression [298,320]. AT-RvDs promoted macrophage-mediated phagocytosis of therapy-generated H460 and HCC827 human lung cancer cells and therapy-killed murine Lewis lung carcinoma (LLC) tumor cells [136]. AT-RvD1, AT-RvD3, and AT- LXA_4_ hindered tumor growth in various murine cancer cell lines, including LLC lung, MC38 colon, and 4T1 breast cells [136]. Furthermore, both AT-LXA_4_ and AT-RvD3 inhibited the release of proinflammatory cytokines, including the macrophage migration inhibitory factor (MIF); MIF secretion decreased due to AT-LXA_4_ or AT-RvD3 treatment of macrophages incubated with etoposide-generated human H460 lung tumor cell debris [136]. Another study suggested the potential for AT-RvDs in preventing tumor development and spread. This study found that AT-RvD1 inhibited the TGF-b1-induced epithelial to mesenchymal transition of A549 human lung adenocarcinoma epithelial cells, thus blocking tumor cell migration and metastasis [137]. In another model of lung adenocarcinoma in mice with an oncogenic *Kras^G12D^* mutation, AT-RvD1 decreased tumor growth and reduced the neutrophil-to-lymphocyte ratio, which is a marker for poor prognosis of cancer outcomes [138].

#### 5.1.3. Protectins and Maresins

To the best of our knowledge, the effects of NSAIDs on protectins and maresins in cancer have not been reported. The synthesis of protectins [321] and maresins (macrophage mediator in resolving inflammation) [322,323] also occurs during the resolution of inflammation. However, their pro-resolving actions may be critical in cancer. 

The 17*R* epimer of PD1 is generated via an aspirin-triggered pathway in which aspirin-acetylated COX-2 of leukocytes catalyzes DHA to 17-*R*-hydroperoxyDHA (17-*R*-HDHA), which undergoes epoxidation to form the 16*R*,17*R*-epoxy-protectin, and then hydrolysis to form aspirin-triggered protectin D1 (AT-PD1/AT-NPD1) [317,321]. PD1 and AT-PD1 inhibit trans-endothelial leukocyte migration and stimulate macrophage-mediated efferocytosis [297,321,324]. Because PD1 partially inhibits the recruitment of leukocytes to the inflamed site, PD1 exerts its anti-inflammatory effects without compromising the body’s immune defense. In addition, the actions of PD1 on various cell types allow for the regulation of cell signaling and transmigration of immune cells during the inflammatory response [297]. 

Maresins, derived from DHA via the 12-LOX pathway, are synthesized by macrophages [297]; the biosynthesis of maresin 1 (MaR1) [323] and maresin 2 (MaR2) [322] have been studied in detail. The bioactions of maresins include antinociception, tissue healing and regeneration, and return to homeostasis, making them critical for host defense and counter-regulators of inflammation [297]. The epoxide intermediate in the maresin biosynthetic pathway, 13,14-epoxyDHA, directly inhibits the enzyme LTA_4_ hydrolase, thus affecting LTB_4_ formation [297,325]. These findings show the role of maresins in regulating proinflammatory lipid mediators in addition to their anti-inflammatory actions, either directly or by way of precursors along the biosynthetic pathways [325]. Maresins prevent PMN infiltration and increase the phagocytic uptake of dead PMNs by macrophages, with comparable potency to RvE1 and PD1 [323]. MaR1 stimulated efferocytosis and tissue regeneration with higher potency than RvD1 and decreased the chemotactic movement of peripheral blood neutrophils [326]. Less potent than MaR1, MaR2 is still effective in promoting efferocytosis and blocking the arrival of neutrophils to the inflamed site, but at lower concentrations [322]. MaR1 possesses anti-inflammatory actions that are also apparent in cancer and metastasis, partly due to its counter-regulation of macrophage-secreted proinflammatory signals and inhibition of tumor growth and metastasis [327]. MaR1 promotes macrophage phagocytotic uptake of chemotherapy-treated and apoptotic human tumor cells of the PC3M-LN prostate, A375-SM melanoma, HEY ovarian, and HSC-3 oral cell lines [327]. These effects were also observed in estrogen-receptor-positive and -negative breast cancer cell lines [328].

## 6. Summary and Perspectives

NSAIDs inhibit the eicosanoid pathway, paving the way for mechanistic drug-developmental work focusing on COX and its products. Overexpression of COX-2 in the colon and many other cancers provided the rationale for clinical trials with COXIBs for cancer prevention or treatment. As alluded to here, the combination of COXIBs with chemotherapeutic agents has been disappointing. NSAIDs do not require the presence of COX-2 to prevent cancer. This review highlights the effects of NSAIDs and COXIBs on targets beyond COX-2 that have shown to be important against many cancers. A significant need in chemoprevention is the development of effective and safe agents. NSAIDs, in general, including COXIBs, do not meet these criteria. Developing the appropriate pharmacological agents and identifying biomarkers that will aid in both monitoring the response and selecting the best candidates for chemoprevention is key to the development of such agents.

There are no “magic bullets” when it comes to cancer treatment. This is a complex disease and, as such, requires a multi-prong, mechanistically targeted approach. Cancer treatment modalities have principally concentrated on the destruction of the tumor cell. Strategies to modulate the host microenvironment offer a new perspective in cancer chemotherapy. How to target cancer-related inflammation in patients with cancer has been a big dilemma. Understanding the various biochemical and signaling pathways involved in tumor cell kinetics has provided the necessary tools for designing agents that augment or inhibit these potential targets.

Aspirin and NSAIDs inhibit the cyclooxygenase pathway, limiting prostanoid biosynthesis. What is important is that aspirin is an irreversible inhibitor that acetylates COX; other NSAIDs are reversible inhibitors. Aspirin acetylation of COX-2 modifies the catalytic domain, blocking PG biosynthesis, but the enzyme remains active, producing 15R-HETE from arachidonic acid, 18R-HEPE from EPA, and 17R-HDHA from DHA in COX-2-expressing cells. Leukocytes transform these to aspirin-triggered lipoxins, aspirin-triggered resolvins, and aspirin-triggered protectins. These AT-SPMs are potent mediators that stop PMN infiltration and enhance macrophage cleanup. Of note, COX-1 does not produce appreciable aspirin-triggered epimers, and NSAIDs inhibit both COX-1 and COX-2, and COXIBs prevent their production. Thus, aspirin-triggered SPMs may induce cancer resolution, demonstrating their utility for chemotherapy. In this arena, much work still needs to be done.

## Figures and Tables

**Table 1:** Selected COX-independent targets of NSAIDs in cancer discussed in the text.

Target	NSAID	Reference
NF-κB	Aspirin	[67,68,69]
	Diclofenac	[70]
	Sulindac	[71,72]
PDK-1/Akt	Celecoxib	[73,74]
	Naproxen	[75]
PPAR	Aspirin	[76]
	Ibuprofen	[76]
	Indomethacin	[76,77]
	Sulindac	[78]
	NS398	[79]
	Celecoxib	[80]
MAPKs	Indomethacin	[81,82,83]
	NS398	[81,84,85]
	Celecoxib	[86,87,88]
	Sulindac sulfide	[85]
Wnt/β-catenin	Indomethacin	[89]
	Sulindac	[90,91,92]
	Diclofenac	[93]
	Celecoxib	[93,94]
PDEs	Sulindac sulfide	[95,96,97,98]
mTOR	Aspirin	[99]
	Celecoxib	[100]
Autophagy	Celecoxib	[101,102,103]
	Meloxicam	[104,105]
	Aspirin	[99,106]
Cell kinetics	Sulindac	[47]
	Piroxicam	[47]
	Celecoxib	[107]
	SC560	[107]
	Naproxen	[75]
	Sulindac sulfide	[108]
Cytochrome c	Indomethacin	[109,110]
	Celecoxib	[111,112]
	NS398	[113]
	Aspirin	[114]
NAG-1	Sulindac	[115]
	Sulindac sulfide	[108,116,117,118]
	Indomethacin	[116,118,119]
	Piroxicam	[119]
	Diclofenac	[119]
	Aspirin	[118]
	Celecoxib	[120,121]
	NS398	[122]
	Ibuprofen, Flurbiprofen	[123]
Ca^2+^ mobilization	Indomethacin	[109,124]
	Celecoxib	[125]
	2,3-Dimethylcelecoxib (DMC, celecoxib analog)	[126,127]
Angiogenesis	Aspirin	[128,129]
	Ibuprofen	[130,131]
Carbonic Anhydrase	Indomethacin	[132]
	Celecoxib	[132,133,134]
	Valdecoxib	[133,134]
SPMs Lipoxins Resolvins Protectins Maresins		
	Aspirin	[135]
	Aspirin	[136,137,138]
	Not studied	
	Not studied	

## Data Availability

Not applicable.

## Abbreviations

13-HDPA	13-hydroxyDPA
13-HODE	13-Hydroxyoctadecadienoic acid
13-HpDPA	13-hydroperoxyDPA
15-HEPE	15-hydroxyeicosapentaenoic acid
15-HETE	15-Hydroxyeicosatetraenoic acid
17-*R*-HDHA	17-*R*-hydroperoxyDHA
18-HEPE	18-hydroxyeicosapentaenoic acid
AA	Arachidonic acid; (20:4, n-6)
Akt	Protein kinase B (PKB)
Apaf-1	Apoptotic protease activating factor 1
APC	Adenomatous polyposis coli
APPROVe	Adenomatous Polyp Prevention on Vioxx
AT-LXA_4_	Aspirin-triggered lipoxin A_4_
ATL	Aspirin-triggered lipoxin
AT-PD1/AT-NPD1	Aspirin-triggered protectin D1/Aspirin-triggered neuroprotectin D1
AT-RvD	Aspirin-triggered D-series resolvin
AT-RvE	Aspirin-triggered E-series resolvin
Bad	Bcl-2-agonist of death
Bak	Bcl-2 homologous antagonist/killer
Bax	Bcl-2-like protein 4
Bcl-2	B-cell lymphoma 2
BH3	Bcl-2 homology 3
Bid	BH3-interacting domain death agonist
Bim	Bcl-2-like protein 11
CA	Carbonic anhydrase
CAI	Carbonic anhydrase inhibitor
cAMP	Cyclic adenosine monophosphate
cdk4	Cyclin-dependent kinase 4
cGMP	Cyclic guanosine monophosphate
CLASS	Celecoxib Long-Term Arthritis Safety Study
CO_2_	Carbon dioxide
COX	Cyclooxygenase
COXIBs	COX-2 selective NSAIDs
DCP	Dichlorophenamide
DHA	Docosahexaenoic acid; (22:6, n-3)
DMC	2,5-Dimethyl-celecoxib
DPA	Docosapentaenoic acid (22:5, n-3)
EGR-1	Early growth response factor 1
EPA	Eicosapentaenoic acid; (20:5, n-3)
ER	Endoplasmic reticulum
ERK	Extracellular-signal-regulated kinase
FAP	Familial adenomatous polyposis
GDP	Guanosine diphosphate
GI	Gastrointestinal
GSK-3β	Glycogen synthase kinase-3beta
GTP	Guanosine triphosphate
IL	Interleukin
IP3	Inositol trisphosphate
IκB	I-kappa-B
JNK	c-Jun N-terminal kinase
LA	Linoleic acid; (18:2, n-6)
LOX	Lipoxygenase
LT	Leukotriene
LTA_4_	Leukotriene A_4_
LX	Lipoxin
MAPK	Mitogen-activated protein kinase
MaR	Maresin
MEK	MAPK/ERK kinase
MIF	migration inhibitory factor
mTOR	Mammalian target of rapamycin
mTORC1	Mammalian target of rapamycin complex 1
mTORC2	Mammalian target of rapamycin complex 2
NAG-1	NSAID-activated gene
NF-κB	Nuclear factor kappa light-chain enhancer of activated B cells
NK cells	Natural killer cells
NSAIDs	Nonsteroidal anti-inflammatory drugs
PD1/NPD1	Protectin D1/Neuroprotectin D1
PDE	Phosphodiesterase
PDK-1	phosphoinositide-dependent kinase-1
PDs	Protectins
PG	Prostaglandin
PGI_2_	Prostacyclin
PI3K	Phosphoinositide 3-kinase
PIP_2_	Phosphatidylinositol (4,5)- bisphosphate
PIP_3_	Phosphatidylinositol (3,4,5)- trisphosphate
PKG	Protein kinase G
PMNs	Polymorphonuclear leukocytes
PPAR	Peroxisome-proliferator-activated receptor
PUFAs	Polyunsaturated fatty acids
Puma	p53 upregulated modulator of apoptosis; Bcl-2-binding component 3
RTX	retinoid X receptor
Rv	Resolvin
RvD	D-series resolvin
RvE	E-series resolvin
RvT	T-series resolvin
SPMs	Specialized pro-resolving mediators
TAM	Tumor-associated macrophages
Tcf-4	Transcription factor 4
TGF-β	Transforming growth factor-beta
TNF-α	Tumor necrosis factor-alpha
TXA_2_	Thromboxane A_2_
VEGF	Vascular endothelial growth factor
VIGOR	Vioxx Gastrointestinal Outcomes Research
Wnt/β-catenin	Wingless-related integration site/beta-catenin

## References

[1] Ley K. (2001). Physiology of Inflammation.

[2] Kashfi K. (2009). Anti-inflammatory agents as cancer therapeutics. Adv. Pharm..

[3] Balkwill F.A. (2001). Mantovani Inflammation and cancer: Back to Virchow?. Lancet.

[4] Medzhitov R. (2008). Origin and physiological roles of inflammation. Nature.

[5] Kumar R., Clermont G., Vodovotz Y., Chow C.C. (2004). The dynamics of acute inflammation. J. Theor. Biol..

[6] Freire O.M., van Dyke T.E. (2013). Natural resolution of inflammation. Periodontol. 2000.

[7] Nathan C., Ding A. (2010). Nonresolving Inflammation. Cell.

[8] Thun M.J., Henley S.J., Patrono C. (2002). Nonsteroidal anti-inflammatory drugs as anticancer agents: Mechanistic pharmacologic and clinical issues. J. Natl. Cancer Inst..

[9] Sandler R.S., Halabi S., Baron J.A., Budinger S., Paskett E., Keresztes R., Petrelli N., Pipas J.M., Karp D.D., Loprinzi C.L. (2003). A randomized trial of aspirin to prevent colorectal adenomas in patients with previous colorectal cancer. N. Engl. J. Med..

[10] Baron J.A., Cole B.F., Sandler R.S., Haile R.W., Ahnen D., Bresalier R., Mckeown-Eyssen G., Summers R.W., Rothstein R., Burke C.A. (2003). A randomized trial of aspirin to prevent colorectal adenomas. N. Engl. J. Med..

[11] Baandrup L., Kjaer S.K., Olsen J.H., Dehlendorff C., Friis S. (2015). Low-dose aspirin use and the risk of ovarian cancer in Denmark. Ann. Oncol..

[12] Trabert B., Ness R.B., Lo-Ciganic W.-H., Murphy M.A., Goode E.L., Poole E.M., Brinton L.A., Webb P.M., Nagle C.M., Jordan S.J. (2014). Aspirin nonaspirin nonsteroidal anti-inflammatory drug and acetaminophen use and risk of invasive epithelial ovarian cancer: A pooled analysis in the Ovarian Cancer Association Consortium. J. Natl. Cancer Inst..

[13] Shebl F.M., Sakoda L.C., Black A., Koshiol J., Andriole G.L., Grubb R., Church T.R., Chia D., Zhou C.K., Chu L.W. (2012). Aspirin but not ibuprofen use is associated with reduced risk of *Prostate* cancer: A PLCO study. Br. J. Cancer.

[14] Sahasrabuddhe V.V., Sahasrabuddhe V.V., Chan A.T., Alavanja M.C., Beane-Freeman L.E., Buring J.E., Chen J., Chong D.Q., Freedman N.D., Fuchs C.S. (2012). Nonsteroidal anti-inflammatory drug use chronic liver disease and hepatocellular carcinoma. J. Natl. Cancer Inst..

[15] Clouser M.C., Roe D.J., Foote J.A., Harrism R.B. (2009). Effect of non-steroidal anti-inflammatory drugs on non-melanoma skin cancer incidence in the SKICAP-AK trial. Pharm. Drug Saf..

[16] Elmets C.A., Viner J.L., Pentland A.P., Cantrell W., Lin H.-Y., Bailey H., Kang S., Linden K.G., Heffernan M., Duvic M. (2010). Chemoprevention of nonmelanoma skin cancer with celecoxib: A randomized double-blind placebo-controlled trial. J. Natl. Cancer Inst..

[17] Sun L., Yu S. (2011). Meta-analysis: Non-steroidal anti-inflammatory drug use and the risk of esophageal squamous *Cell* carcinoma. Dis. Esophagus.

[18] Cui X.J., He Q., Zhang J.-M., Fan H.-J., Wen Z.-F., Qin Y.-R. (2014). High-dose aspirin consumption contributes to decreased risk for pancreatic cancer in a systematic review and meta-analysis. Pancreas.

[19] Yiannakopoulou E. (2015). Aspirin and NSAIDs for breast cancer chemoprevention. Eur. J. Cancer Prev..

[20] de Pedro M., Baeza S., Escudero M.T., Dierssen-Sotos T., Gómez-Acebo I., Pollán M., Llorca J. (2015). Effect of COX-2 inhibitors and other non-steroidal inflammatory drugs on breast cancer risk: A meta-analysis. Breast Cancer Res. Treat..

[21] Daugherty S.E., Pfeiffer R.M., Sigurdson A.J., Hayes R.B., Leitzmann M., Schatzkin A., Hollenbeck A.R., Silverman D.T. (2011). Nonsteroidal antiinflammatory drugs and bladder cancer: A pooled analysis. Am. J. Epidemiol..

[22] Nicastro H.L., Grubbs C.J., Juliana M.M., Bode A.M., Kim M.-S., Lu Y., You M., Milne G., Boring D., Steele V.E. (2014). Preventive effects of NSAIDs NO-NSAIDs and NSAIDs plus difluoromethylornithine in a chemically induced urinary bladder cancer model. Cancer Prev. Res..

[23] Becker C., Wilson J.C., Jick S.S., Meier C.R. (2015). Non-steroidal anti-inflammatory drugs and the risk of head and neck cancer: A case-control analysis. Int. J. Cancer.

[24] Rothwell P.M., Price J.F., Fowkes F.G.R., Zanchetti A., Roncaglioni M.C., Tognoni G., Lee R., Belch J.J., Wilson M., Mehta Z. (2012). Short-term effects of daily aspirin on cancer incidence mortality and non-vascular death: Analysis of the time course of risks and benefits in 51 randomised controlled trials. Lancet.

[25] Algra A.M., Rothwell P.M. (2012). Effects of regular aspirin on long-term cancer incidence and metastasis: A systematic comparison of evidence from observational studies versus randomised trials. Lancet Oncol..

[26] Simmons D.L., Botting R.M., Hla T. (2004). Cyclooxygenase Isozymes: The Biology of Prostaglandin Synthesis and Inhibition. Pharm. Rev..

[27] Brunton L., Knollman B., Hilal-Dandan R. (2017). Goodman and Gilman’s the Pharmacological Basis of Therapeutics.

[28] Booker R. (2013). NSAIDs: Uses effects risks and benefits. Pract. Nurse.

[29] Gryglewski R.J., Dembínska-Kieć A., Korbut R. (1978). A possible role of thromboxane A2 (TXA2) and prostacyclin (PGI2) in circulation. Acta Biol. Med. Ger..

[30] Fiorucci S., Antonelli E. (2001). Cyclo-oxygenase isoenzymes. Structural basis for selective inhibition of cyclo-oxygenases by anti-inflammatory agents. Dig. Liver Dis..

[31] Kune G., Kune S., Watson L. (1988). Colorectal cancer risk chronic illnesses operations and medications: Case-control results from the Melbourne Colorectal Cancer Study. Cancer Res..

[32] Ashok V., Dash C., Rohan T.E., Sprafka J.M., Terry P.D. (2011). Selective cyclooxygenase-2 (COX-2) inhibitors and breast cancer risk. Breast.

[33] Rozic J.G., Chakraborty C., Lala P.K. (2001). Cyclooxygenase inhibitors retard murine mammary tumor progression by reducing tumor *Cell* migration invasiveness and angiogenesis. Int. J. Cancer.

[34] Xu L., Stevens J., Hilton M.B., Seaman S., Conrads T.P., Veenstra T.D., Logsdon D., Morris H., Swing D.A., Patel N.L. (2014). COX-2 Inhibition Potentiates Antiangiogenic Cancer Therapy and Prevents Metastasis in Preclinical Models. Sci. Trans. Med..

[35] Barnard M.E., Poole E.M., Curhan G.C., Eliassen A.H., Rosner B.A., Terry K.L., Tworoger S.S. (2018). Association of Analgesic Use With Risk of Ovarian Cancer in the Nurses’ Health Studies. JAMA Oncol..

[36] Bjorkman D.J. (1999). Current status of nonsteroidal anti-inflammatory drug (NSAID) use in the United States: Risk factors and frequency of complications. Am. J. Med..

[37] Wallace J.L. (2008). Prostaglandins NSAIDs and gastric mucosal protection: Why doesn’t the stomach digest itself?. Physiol. Rev..

[38] Kashfi K., Rigas B. (2005). Non-COX-2 targets and cancer: Expanding the molecular target repertoire of chemoprevention. Biochem. Pharm..

[39] Bennett A., Civier A., Hensby C.N., Melhuish P.B., Stamford I.F. (1987). Measurement of arachidonate and its metabolites extracted from human normal and malignant gastrointestinal tissues. Gut.

[40] Rigas B., Goldman I.S., Levine L. (1993). Altered eicosanoid levels in human colon cancer. J. Lab. Clin. Med..

[41] Eberhart C.E., Coffey R.J., Radhika A., Giardiello F.M., Ferrenbach S., Dubois R.N. (1994). Up-regulation of cyclooxygenase 2 gene expression in human colorectal adenomas and carcinomas. Gastroenterology.

[42] Wu Q.-B., Sun G.-P. (2015). Expression of COX-2 and HER-2 in colorectal cancer and their correlation. World J. Gastroenterol. Gastroenterol..

[43] Sheng J., Sun H., Yu F.-B., Li B., Zhang Y., Zhu Y.-T. (2020). The Role of Cyclooxygenase-2 in Colorectal Cancer. Int. J. Med. Sci..

[44] Dannenberg A.J., Subbaramaiah K. (2003). Targeting cyclooxygenase-2 in human neoplasia: Rationale and promise. Cancer Cancer Cell Cell.

[45] Oshima M., Dinchuk J.E., Kargman S.L., Oshima H., Hancock B., Kwong E., Trzaskos J.M., Evans J.F., Taketo M.M. (1996). Suppression of intestinal polyposis in Apc^Ð716^ knockout mice by inhibition of cyclooxygenase 2 (COX-2). Cell.

[46] North G.L. (2001). Celecoxib as adjunctive therapy for treatment of colorectal cancer. Ann. Pharm..

[47] Hanif R., Pittas A., Feng Y., Koutsos M.I., Qiao L., Staiano-Coico L., Shiff S.I., Rigas B. (1996). Effects of nonsteroidal anti-inflammatory drugs on proliferation and on induction of apoptosis in colon cancer *Cells* by a prostaglandin-independent pathway. Biochem. Pharm..

[48] Elder D.J.E., Halton D.E., Hague A., Paraskeva C. (1997). Induction of apoptotic cell death in human colorectal carcinoma *Cell* lines by a cyclooxygenase-2 (COX-2)-selective nonsteroidal anti-inflammatory drug—Independence from COX-2 protein expression. Clin. Cancer Res..

[49] Dang C.T., Dannenberg A.J., Subbaramaiah K., Dickler M.N., Moasser M.M., Seidman A.D., D’Andrea G.M., Theodoulou M. (2004). Phase II study of celecoxib and trastuzumab in metastatic breast cancer patients who have progressed after prior trastuzumab-based treatments. Clin. Cancer Res..

[50] Becerra C.R., Frenkel E.P., Ashfaq R., Gaynor R.B. (2003). Increased toxicity and lack of efficacy of Rofecoxib in combination with chemotherapy for treatment of metastatic colorectal cancer: A phase II study. Int. J. Cancer.

[51] Park A., Lee Y., Kim M.S., Kang Y.J., Park Y.J., Jung H., Kim T.D., Lee H.G., Choi I., Yoon S.R. (2018). Prostaglandin E2 Secreted by Thyroid Cancer *Cells* Contributes to Immune Escape Through the Suppression of Natural Killer (NK) *Cell* Cytotoxicity and NK *Cell* Differentiation. Front. Immunol..

[52] Bacchi S., Palumbo P., Sponta A., Coppolino M.F. (2012). Clinical pharmacology of non-steroidal anti-inflammatory drugs: A review. Antiinflamm. Antiallergy Agents Med. Chem..

[53] Fiorucci S. (2009). Prevention of nonsteroidal anti-inflammatory drug-induced ulcer: Looking to the future. Gastroenterol. Clin. N. Am..

[54] Wallace J.L., Viappiani S., Bolla M. (2009). Cyclooxygenase-inhibiting nitric oxide donators for osteoarthritis. Trends Pharm. Sci..

[55] Chinthapalli K. (2013). High dose NSAIDs may double the risk of heart attacks and heart failure says new study. BMJ.

[56] Fiorucci S., Distrutti E. (2011). COXIBs CINODs and HS-releasing NSAIDs: Current perspectives in the development of safer non steroidal anti-inflammatory drugs. Curr. Med. Chem..

[57] Pepine C.J., Gurbel P.A. (2017). Cardiovascular safety of NSAIDs: Additional insights after PRECISION and point of view. Clin. Cardiol..

[58] Walker C., Biasucci L.M. (2018). Cardiovascular safety of non-steroidal anti-inflammatory drugs revisited. Postgrad. Med..

[59] Bombardier C., Laine L., Reicin A., Shapiro D., Burgos-Vargas R., Davis B., Day R., Ferraz M.B., Hawkey C.J., Hochberg M.C. (2000). Comparison of upper gastrointestinal toxicity of rofecoxib and naproxen in patients with rheumatoid arthritis. VIGOR Study Group. N. Engl. J. Med..

[60] Silverstein F.E., Faich G., Goldstein J.L., Simon L.S., Pincus T., Whelton A., Makuch R., Eisen G., Agrawal N.M., Stenson W.F. (2000). Gastrointestinal toxicity with celecoxib vs. nonsteroidal anti-inflammatory drugs for osteoarthritis and rheumatoid arthritis: The CLASS study: A randomized controlled trial. Celecoxib Long-term Arthritis Safety Study. JAMA.

[61] Inotai A., Mészáros A. (2009). Economic evaluation of nonsteroidal anti-inflammatory drug strategies in rheumatoid arthritis. Int. J. Technol. Assess Health Care.

[62] Chen L.C., Ashcroft D.M. (2007). Risk of myocardial infarction associated with selective COX-2 inhibitors: Meta-analysis of randomised controlled trials. Pharm. Drug Saf..

[63] Bresalier R.S., Sandler R.S., Quan H., Bolognese J.A., Oxenius B., Horgan K., Lines C., Riddell R., Morton D., Lanas A. (2005). Cardiovascular events associated with rofecoxib in a colorectal adenoma chemoprevention trial. N. Engl. J. Med..

[64] FitzGerald G.A., Patrono C. (2001). The coxibs selective inhibitors of cyclooxygenase-2. N. Engl. J. Med..

[65] Singh G., Miller J.D., Huse D.M., Pettitt D., D’Agostino R.B., Russell M.W. (2003). Consequences of increased systolic blood pressure in patients with osteoarthritis and rheumatoid arthritis. J. Rheumatol..

[66] Kearney P.M., Baigent C., Godwin J., Halls H., Emberson J.R., Patrono C. (2006). Do selective cyclo-oxygenase-2 inhibitors and traditional non-steroidal anti-inflammatory drugs increase the risk of atherothrombosis? Meta-analysis of randomised trials. BMJ.

[67] Park M.H., Hong J.T. (2016). Roles of NF-κB in cancer and inflammatory diseases and their therapeutic approaches. Cells.

[68] Kopp E., Ghosh S. (1994). Inhibition of NF-kappa B by sodium salicylate and aspirin. Science.

[69] Stark L.A., Din F.V.N., Zwacka R.M., Dunlop M.G. (2001). Aspirin-induced activation of the NF-kappaB signaling pathway: A novel mechanism for aspirin-mediated apoptosis in colon cancer cells. FASEB J..

[70] Cho M., Gwak J., Park S., Won J., Kim D.E., Yea S.S., Cha I.J., Kim T.K., Shin J.G., Oh S. (2005). Diclofenac attenuates Wnt/β-catenin signaling in colon cancer cells by activation of NF-κB. FEBS Lett..

[71] Yamamoto Y., Gaynor R.B. (2001). Role of the NF-kappaB pathway in the pathogenesis of human disease states. Mol. Med. Curr. Mol. Med..

[72] Yamamoto Y., Yin M.J., Lin K.M., Gaynor R. (1999). BSulindac inhibits activation of the NF-kappaB pathway. J. Biol. Chem..

[73] Arico S., Pattingre S., Bauvy C., Gane P., Barbat A., Codogno P., Ogier-Denis E. (2002). Celecoxib induces apoptosis by inhibiting 3-phosphoinositide-dependent protein kinase-1 activity in the human colon cancer HT-29 cell line. J. Biol. Chem..

[74] Benelli R., Barboro P., Costa D., Astigiano S., Barbieri O., Capaia M., Poggi A., Ferrari N. (2019). Multifocal signal modulation therapy by celecoxib: A strategy for managing castration-resistant prostate cancer. Int. J. Mol. Sci..

[75] Kim M.S., Kim J.E., Huang Z., Chen H., Langfald A., Lubet R.A., Grubbs C.J., Dong Z., Bode A.M. (2014). Naproxen Induces Cell-Cycle Arrest and Apoptosis in Human Urinary Bladder *Cancer Cell* Lines and Chemically Induced Cancers by Targeting PI3K. Cancer Prev. Res..

[76] Lehmann J.M., Lenhard J.M., Oliver B.B., Ringold G.M., Kliewer S.A. (1997). Peroxisome proliferator-activated receptors α and γ are activated by indomethacin and other non-steroidal anti-inflammatory drugs. J. Biol. Chem..

[77] He T.-C., Chan T.A., Vogelstein B., Kinzler K.W. (1999). PPARδ Is an APC-regulated target of nonsteroidal anti-inflammatory drugs. Cell.

[78] Park B.H., Vogelstein B., Kinzler K.W. (2001). Genetic disruption of Genetic disruption of PPARδ decreases the tumorigenicity of human colon cancer cells. Proc. Natl. Acad. Sci. USA.

[79] Sun W.H., Chen G.S., Ou X.L., Yang Y., Luo C., Zhang Y., Shao Y., Xu H.C., Xiao B., Xue Y.P. (2009). Inhibition of COX-2 and activation of peroxisome proliferator-activated receptor gamma synergistically inhibits proliferation and induces apoptosis of human pancreatic carcinoma cells. Cancer Lett..

[80] Narayanan N.K., Narayanan B.A., Reddy B.S. (2005). A combination of docosahexaenoic acid and celecoxib prevents prostate cancer cell growth in vitro and is associated with modulation of nuclear factor-kappaB and steroid hormone receptors. Int. J. Oncol..

[81] Husain S.S., Szabo I.L., Pai R., Soreghan B., Jones M.K., Tarnawski A.S. (2001). MAPK (ERK2) kinase—A key target for NSAIDs-induced inhibition of gastric cancer cell proliferation and growth. Life Sci..

[82] Ou Y.C., Yang C.R., Cheng C.L., Raung S.L., Hung Y.Y., Chen C.J. (2007). Indomethacin induces apoptosis in 786-O renal cell carcinoma cells by activating mitogen-activated protein kinases and AKT. Eur. J. Pharmacol..

[83] Kim T., Jin S., Kim W., Kang E., Choi K., Kim H., Shin S., Kang J. (2001). Prolonged activation of mitogen-activated protein kinases during NSAID-induced apoptosis in HT-29 colon cancer cells. Int. J. Colorectal Dis..

[84] Elder D.J.E., Halton D.E., Playle L.C., Paraskeva C. (2002). The MEK/ERK pathway mediates COX-2-selective NSAID-induced apoptosis and induced COX-2 protein expression in colorectal carcinoma cells. Int. J. Cancer.

[85] Sun Y., Sinicrope F.A. (2005). Selective inhibitors of MEK1/ERK^44/42^ and p38 mitogen-activated protein kinases potentiate apoptosis induction by sulindac sulfide in human colon carcinoma cells. Mol. Cancer Therap..

[86] Setia S., Nehru B., Sanyal S.N. (2014). Upregulation of MAPK/Erk and PI3K/Akt pathways in ulcerative colitis-associated colon cancer. Biomed. Pharm..

[87] Jia Z., Zhang H., Ma C., Li N., Wang M. (2021). Celecoxib enhances apoptosis of the liver cancer cells via regulating ERK/JNK/P38 pathway. J. Buon.

[88] Park S.W., Kim H.S., Hah J.W., Jeong W.J., Kim K.H., Sung M.W. (2010). Celecoxib inhibits cell proliferation through the activation of ERK and p38 MAPK in head and neck squamous cell carcinoma cell lines. Anticancer Drugs.

[89] Heidel Florian H., Bullinger L., Feng Z., Wang Z., Neff T.A., Stein L., Kalaitzidis D., Lane S.W., Armstrong S.A. (2012). Genetic and pharmacologic inhibition of β-catenin targets imatinib-resistant leukemia stem cells in CML. Cell Stem Cell.

[90] Han A., Song Z., Tong C., Hu D., Bi X., Augenlicht L.H., Yang W. (2008). Sulindac suppresses β-catenin expression in human cancer cells. Eur. J. Pharm..

[91] Rice P.L., Kelloff J., Sullivan H., Driggers L.J., Beard K.S., Kuwada S., Piazza G., Ahnen D.J. (2003). Sulindac metabolites induce caspase- and proteasome-dependent degradation of β-catenin protein in human colon cancer cells. Mol. Cancer Therap..

[92] Boon E.M.J., Keller J.J., Wormhoudt T.A.M., Giardiello F.M., Offerhaus G.J.A., Van Der Neut R., Pals S.T. (2004). Sulindac targets nuclear β-catenin accumulation and Wnt signalling in adenomas of patients with familial adenomatous polyposis and in human colorectal cancer cell lines. Br. J. Cancer.

[93] Sareddy G.R., Kesanakurti D., Kirti P.B., Babu P.P. (2013). Nonsteroidal anti-inflammatory drugs diclofenac and celecoxib attenuates Wnt/β-catenin/Tcf signaling pathway in human glioblastoma cells. Neurochem. Res..

[94] Huang C., Chen Y., Liu H., Yang J., Song X., Zhao J., He N., Zhou C.J., Wang Y., Huang C. (2017). Celecoxib targets breast cancer stem cells by inhibiting the synthesis of prostaglandin E and down-regulating the Wnt pathway activity. Oncotarget.

[95] Tinsley H.N., Gary B.D., Keeton A.B., Zhang W., Abadi A.H., Reynolds R.C., Piazza G.A. (2009). Sulindac sulfide selectively inhibits growth and induces apoptosis of human breast tumor cells by phosphodiesterase 5 inhibition elevation of cyclic GMP and activation of protein kinase G. Mol. Cancer Therap..

[96] Tinsley H.N., Gary B.D., Thaiparambil J., Li N., Lu W., Li Y., Maxuitenko Y.Y., Keeton A.B., Piazza G.A. (2010). Colon tumor cell growth-inhibitory activity of sulindac sulfide and other nonsteroidal anti-inflammatory drugs is associated with phosphodiesterase 5 inhibition. Cancer Prev. Res..

[97] Tinsley H.N., Gary B.D., Keeton A.B., Lu W., Li Y., Piazza G.A. (2011). Inhibition of PDE5 by sulindac sulfide selectively induces apoptosis and attenuates oncogenic Wnt/β-catenin-mediated transcription in human breast tumor cells. Cancer Prev. Res..

[98] Li N., Xi Y., Tinsley H.N., Gurpinar E., Gary B.D., Zhu B., Li Y., Chen X., Keeton A.B., Abadi A.H. (2013). Sulindac selectively inhibits colon tumor cell growth by activating the cGMP/PKG pathway to suppress Wnt/β-catenin signaling. Mol. Cancer Therap..

[99] Din F.V., Valanciute A., Houde V.P., Zibrova D., Green K.A., Sakamoto K., Alessi D.R., Dunlop M.G. (2012). Aspirin inhibits mTOR signaling activates amp-activated protein kinase and induces autophagy in colorectal cancer cells. Gastroenterology.

[100] Zhang P., He D., Song E., Jiang M., Song Y. (2019). Celecoxib enhances the sensitivity of non-small-cell lung cancer *Cells* to radiation-induced apoptosis through downregulation of the Akt/mTOR signaling pathway and COX-2 expression. PLoS ONE.

[101] Lu Y., Liu X.F., Liu T.R., Fan R.F., Xu Y.C., Zhang X.Z., Liu L.L. (2016). Celecoxib exerts antitumor effects in HL-60 acute leukemia cells and inhibits autophagy by affecting lysosome function. Biomed. Pharm..

[102] Huang S., Sinicrope F.A. (2010). Celecoxib-induced apoptosis is enhanced by ABT-737 and by inhibition of autophagy in human colorectal cancer cells. Autophagy.

[103] Huang K.H., Kuo K.L., Ho I.L., Chang H.C., Chuang Y.T., Lin W.C., Lee P.Y., Chang S.C., Chiang C.K., Pu Y.S. (2013). Celecoxib-induced cytotoxic effect is potentiated by inhibition of autophagy in human urothelial carcinoma cells. PLoS ONE.

[104] Zhong J., Dong X., Xiu P., Wang F., Liu J., Wei H., Xu Z., Liu F., Li T., Li J. (2015). Blocking autophagy enhances meloxicam lethality to hepatocellular carcinoma by promotion of endoplasmic reticulum stress. Cell Prolif..

[105] Dong X., Li R., Xiu P., Dong X., Xu Z., Zhai B., Liu F., Jiang H., Sun X., Li J. (2014). Meloxicam executes its antitumor effects against hepatocellular carcinoma in COX-2- dependent and -independent pathways. PLoS ONE.

[106] Zhang C., Shi J., Mao S.Y., Xu Y.S., Zhang D., Feng L.Y., Zhang B., Yan Y.Y., Wang S.C., Pan J.P. (2015). Role of p38 MAPK in enhanced human cancer cells killing by the combination of aspirin and ABT-737. J. Cell Mol. Med..

[107] Grösch S., Tegeder I., Niederberger E., Bräutigam L., Geisslinger G. (2001). COX-2 independent induction of cell cycle arrest and apoptosis in colon cancer cells by the selective COX-2 inhibitor celecoxib. FASEB J..

[108] Kim J.S., Baek S.J., Sali T., Eling T.E. (2005). The conventional nonsteroidal anti-inflammatory drug sulindac sulfide arrests ovarian cancer cell growth via the expression of NAG-1/MIC-1/GDF-15. Mol. Cancer Therap..

[109] Carrasco-Pozo C., Pastene E., Vergara C., Zapata M., Sandoval C., Gotteland M. (2012). Stimulation of cytosolic and mitochondrial calcium mobilization by indomethacin in Caco-2 cells: Modulation by the polyphenols quercetin resveratrol and rutin. Biochim. Biophys. Acta (Bba)—Gen. Subj..

[110] Aggarwal S., Taneja N., Lin L., Orringer M.B., Rehemtulla A., Beer D.G. (2000). Indomethacin-Induced Apoptosis in Esophageal Adenocarcinoma Cells Involves Upregulation of Bax and Translocation of Mitochondrial Cytochrome C Independent of COX-2 Expression. Neoplasia.

[111] Maier T.J., Schilling K., Schmidt R., Geisslinger G., Grösch S. (2004). Cyclooxygenase-2 (COX-2)-dependent and -independent anticarcinogenic effects of celecoxib in human colon carcinoma cells. Biochem. Pharm..

[112] Wang Y.J., Niu X.P., Yang L., Han Z., Ma Y.J. (2013). Effects of celecoxib on cycle kinetics of gastric cancer cells and protein expression of cytochrome C and caspase-9. Asian Pac. J. Cancer Prev..

[113] Li M., Wu X., Xu X.-C. (2001). Induction of apoptosis in colon cancer cells by cyclooxygenase-2 inhibitor NS398 through a cytochrome c-dependent pathway. Clinical Cancer Res..

[114] Zimmermann K.C., Waterhouse N.J., Goldstein J.C., Schuler M., Green D.R. (2000). Aspirin induces apoptosis through release of cytochrome c from mitochondria. Neoplasia.

[115] Kim K.S., Baek S.J., Flake G.P., Loftin C.D., Calvo B.F., Eling T.E. (2002). Expression and regulation of nonsteroidal anti-inflammatory drug–activated gene (NAG-1) in human and mouse tissue. Gastroenterology.

[116] Baek S.J., Kim K.S., Nixon J.B., Wilson L.C., Eling T.E. (2001). Cyclooxygenase inhibitors regulate the expression of a TGF-β superfamily member that has proapoptotic and antitumorigenic activities. Mol. Pharm..

[117] Kadowaki M., Yoshioka H., Kamitani H., Watanabe T., Wade P.A., Eling T.E. (2012). DNA methylation-mediated silencing of nonsteroidal anti-inflammatory drug-activated gene (NAG-1/GDF15) in glioma cell lines. Int. J. Cancer.

[118] Jang T.J., Kang H.J., Kim J.R., Yang C.H. (2004). Non-steroidal anti-inflammatory drug activated gene (NAG-1) expression is closely related to death receptor-4 and -5 induction which may explain sulindac sulfide induced gastric Cancer Cell apoptosis. Carcinogenesis.

[119] Baek S.J., Wilson L.C., Lee C.H., Eling T.E. (2002). Dual function of nonsteroidal anti-inflammatory drugs (nsaids): Inhibition of cyclooxygenase and induction of NSAID-activated gene. J. Pharm. Exp. Therap..

[120] Huang M.T., Chen Z.X., Wei B., Zhang B., Wang C.H., Huang M.H., Liu R., Tang C.W. (2007). Preoperative growth inhibition of human gastric adenocarcinoma treated with a combination of celecoxib and octreotide. Acta Pharm. Sin..

[121] Iguchi G., Chrysovergis K., Lee S.H., Baek S.J., Langenbach R., Eling T.E. (2009). A reciprocal relationship exists between non-steroidal anti-inflammatory drug-activated gene-1 (NAG-1) and cyclooxygenase-2. Cancer Lett..

[122] Youns M., Efferth T., Hoheisel J.D. (2011). Transcript profiling identifies novel key players mediating the growth inhibitory effect of NS-398 on human pancreatic cancer cells. Eur. J. Pharm..

[123] Wynne S., Djakiew D. (2010). NSAID Inhibition of Prostate Cancer Cell Migration Is Mediated by Nag-1 Induction via the p38 MAPK-p75^NTR^ Pathway. Molecular Cancer Res..

[124] Guo Y.C., Chang C.M., Hsu W.L., Chiu S.J., Tsai Y.T., Chou Y.H., Hou M.F., Wang J.Y., Lee M.H., Tsai K.L. (2013). Indomethacin inhibits cancer cell migration via attenuation of cellular calcium mobilization. Molecules.

[125] Johnson A.J., Hsu A.L., Lin H.P., Song X., Chen C.S. (2002). The cyclo-oxygenase-2 inhibitor celecoxib perturbs intracellular calcium by inhibiting endoplasmic reticulum Ca^2+^-ATPases: A plausible link with its anti-tumour effect and cardiovascular risks. Biochem. J..

[126] Pyrko P., Kardosh A., Liu Y.T., Soriano N., Xiong W., Chow R.H., Uddin J., Petasis N.A., Mircheff A.K., Farley R.A. (2007). Calcium-activated endoplasmic reticulum stress as a major component of tumor cell death induced by 2,5-dimethyl-celecoxib a non-coxib analogue of celecoxib. Mol. Cancer Therap..

[127] Coca R., Soler F., Cortes-Castell E., Gil-Guillen V., Fernandez-Belda F. (2014). Inhibition mechanism of the intracellular transporter Ca^2+^-Pump from sarco-endoplasmic reticulum by the antitumor agent dimethyl-celecoxib. PLoS ONE.

[128] Dai X., Yan J., Fu X., Pan Q., Sun D., Xu Y., Wang J., Nie L., Tong L., Shen A. (2017). Aspirin inhibits cancer metastasis and angiogenesis via targeting heparanase. Clin. Cancer Res..

[129] Maity G., Chakraborty J., Ghosh A., Haque I., Banerjee S., Banerjee S.K. (2019). Aspirin suppresses tumor cell-induced angiogenesis and their incongruity. J. Cell Commun. Signal..

[130] Akrami H., Aminzadeh S., Fallahi H. (2015). Inhibitory effect of ibuprofen on tumor survival and angiogenesis in gastric cancer cell. Tumor Biol..

[131] Yao M., Zhou W., Sangha S., Albert A., Chang A.J., Liu T.C., Wolfe M.M. (2005). Effects of nonselective cyclooxygenase inhibition with low-dose ibuprofen on tumor growth angiogenesis metastasis and survival in a mouse model of colorectal cancer. Clin. Cancer Res..

[132] Vlad A.M., Colţău M.I., Pușcaș I. (2015). Relationship between NSAIDs gastric antisecretory and carbonic anhydrase isoenzyme. Acta Med. Trans..

[133] Weber A., Casini A., Heine A., Kuhn D., Supuran C.T., Scozzafava A., Klebe G. (2004). Unexpected nanomolar inhibition of carbonic anhydrase by COX-2-selective celecoxib:  New pharmacological opportunities due to related binding site recognition. J. Med. Chem..

[134] Knudsen J.F., Carlsson U., Hammarström P., Sokol G.H., Cantilena L.R. (2004). the cyclooxygenase-2 inhibitor celecoxib is a potent inhibitor of human carbonic anhydrase II. Inflammation.

[135] Clària J., Lee M.H., Serhan C.N. (1996). Aspirin-triggered lipoxins (15-epi-LX) are generated by the human lung adenocarcinoma cell line (A549)-neutrophil interactions and are potent inhibitors of cell proliferation. Mol. Med..

[136] Gilligan M.M., Gartung A., Sulciner M.L., Norris P.C., Sukhatme V.P., Bielenberg D.R., Huang S., Kieran M.W., Serhan C.N., Panigrahy D. (2019). Aspirin-triggered proresolving mediators stimulate resolution in cancer. Proc. Natl. Acad. Sci. USA.

[137] Liu Y., Yuan X., Li W., Cao Q., Shu Y. (2016). Aspirin-triggered resolvin D1 inhibits TGF-β1-induced EMT through the inhibition of the mTOR pathway by reducing the expression of PKM2 and is closely linked to oxidative stress. Int. J. Mol. Med..

[138] Vannitamby A., Saad M.I., Aloe C., Wang H., Kumar B., Vlahos R., Selemidis S., Irving L., Steinfort D., Jenkins B.J. (2021). Aspirin-triggered resolvin D1 reduces proliferation and the neutrophil to lymphocyte ratio in a mutant KRAS-driven lung adenocarcinoma model. Cancers.

[139] Lawrence T., Gilroy D.W., Colville-Nash P.R., Willoughby D.A. (2001). Possible new role for NF-κB in the resolution of inflammation. Nat. Med..

[140] Huang H.Y., Zhang Z.J., Cao C.B., Wang N., Liu F.F., Peng J.Q., Ren X.J., Qian J. (2014). The TLR4/NF-κB signaling pathway mediates the growth of colon cancer. Eur. Rev. Med. Pharm. Sci..

[141] Nomura A., Majumder K., Giri B., Dauer P., Dudeja V., Roy S., Banerjee S., Saluja A.K. (2016). Inhibition of NF-kappa B pathway leads to deregulation of epithelial–mesenchymal transition and neural invasion in pancreatic cancer. Lab. Investig..

[142] Pires B.R.B., Mencalha A.L., Ferreira G.M., de Souza W.F., Morgado-Díaz J.A., Maia A.M., Corrêa S., Abdelhay E.S. (2017). NF-kappaB is involved in the regulation of EMT genes in breast cancer cells. PLoS ONE.

[143] Annunziata C.M., Stavnes H.T., Kleinberg L., Berner A., Hernandez L.F., Birrer M.J., Steinberg S.M., Davidson B., Kohn E.C. (2010). Nuclear factor κB transcription factors are coexpressed and convey a poor outcome in ovarian cancer. Cancer.

[144] Chen Charlie D., Sawyers L. (2002). Charles NF-κB activates prostate-specific antigen expression and is upregulated in androgen-independent prostate cancer. Mol. Cell. Biol..

[145] Ismail A H., Lessard L., Mes-Masson A.M., Saad F. (2004). Expression of NF-κB in prostate cancer lymph node metastases. Prostate.

[146] Nishio H., Yaguchi T., Sugiyama J., Sumimoto H., Umezawa K., Iwata T., Susumu N., Fujii T., Kawamura N., Kobayashi A. (2014). Immunosuppression through constitutively activated NF-κB signalling in human ovarian cancer and its reversal by an NF-κB inhibitor. Br. J. Cancer.

[147] Longnecker D.S., Wiebkin P., Schaeffer B.K., Roebuck B.D. (1984). Experimental carcinogenesis in the pancreas. Int. Rev. Exp. Pathol..

[148] Kaltschmidt B., Greiner J.F., Kadhim H.M., Kaltschmidt C. (2018). Subunit-Specific Role of NF-κB in Cancer. Biomedicines.

[149] Naugler W.E., Karin M. (2008). NF-κB and cancer—Identifying targets and mechanisms. Curr. Opin. Genet. Dev..

[150] Chen L., Chen L., Deng H., Cui H., Fang J., Zuo Z., Deng J., Li Y., Wang X., Zhao L. (2018). Inflammatory responses and inflammation-associated diseases in organs. Oncotarget.

[151] Hoesel B., Schmid J.A. (2013). The complexity of NF-κB signaling in inflammation and cancer. Mol. Cancer.

[152] Sethi G., Sung B., Aggarwal B.B. (2008). TNF: A master switch for inflammation to cancer. Front. Biosci..

[153] Li Q., Withoff S., Verma I.M. (2005). Inflammation-associated cancer: NF-κB is the lynchpin. Trends Immunol..

[154] Wu Y., Zhou B.P. (2010). TNF-α/NF-κB/Snail pathway in cancer cell migration and invasion. Br. J. Cancer.

[155] Wang G., Li J., Zhang L., Huang S., Zhao X., Zhao X. (2017). Celecoxib induced apoptosis against different breast cancer cell lines by down-regulated NF-κB pathway. Biochem. Biophys. Res. Commun..

[156] Hemmings B.A., Restuccia D.F. (2012). Pi3k-PKB/AKT pathway. Cold Spring Harbor Perspect. Biol..

[157] Nitulescu G.M., Van De Venter M., Nitulescu G., Ungurianu A., Juzenas P., Peng Q., Olaru O.T., Grădinaru D., Tsatsakis A., Tsoukalas D. (2018). The Akt pathway in oncology therapy and beyond. Int. J. Oncol..

[158] Wang Q., Chen X., Hay N. (2017). Akt as a target for cancer therapy: More is not always better (lessons from studies in mice). Br. J. Cancer.

[159] Chalhoub N., Baker S.J. (2009). PTEN and the PI3-kinase pathway in cancer. Annu. Rev. Pathol..

[160] Nakatani K., Thompson D.A., Barthel A., Sakaue H., Liu W., Weigel R.J., Roth R.A. (1999). Up-regulation of Akt3 in estrogen receptor-deficient breast cancers and androgen-independent *Prostate* cancer lines. J. Biol. Chem..

[161] Cristiano B.E., Chan J.C., Hannan K.M., Lundie N.A., Marmy-Conus N.J., Campbell I.G., Phillips W.A., Robbie M., Hannan R.D., Pearson R.B. (2006). A specific role for AKT3 in the genesis of ovarian cancer through modulation of G-M phase transition. Cancer Res..

[162] Osaki M., Oshimura M., Ito H. (2004). PI3K-Akt pathway: Its functions and alterations in human cancer. Apoptosis.

[163] Hennessy B.T., Smith D.L., Ram P.T., Lu Y., Mills G.B. (2005). Exploiting the PI3K/AKT pathway for cancer drug discovery. Nat. Rev. Drug Discov..

[164] Hou C.C., Hung S.L., Kao S.H., Chen T.H., Lee H.M. (2005). Celecoxib induces heme-oxygenase expression in glomerular mesangial cells. Ann. New York Acad. Sci..

[165] Chen Z., Wang C., Dong H., Wang X., Gao F., Zhang S., Zhang X. (2020). Aspirin has a better effect on PIK3CA mutant colorectal cancer cells by PI3K/Akt/raptor pathway. Mol. Med..

[166] Berger J., Moller D.E. (2002). The mechanisms of action of PPARs. Ann. Rev. Med..

[167] Michalik L., Auwerx J., Berger J.P., Chatterjee V.K., Glass C.K., Gonzalez F.J., Grimaldi P.A., Kadowaki T., Lazar M.A., O’Rahilly S. (2006). International Union of Pharmacology. LXI. Peroxisome Proliferator-Activated Receptors. Pharm. Rev..

[168] Sarraf P., Mueller E., Jones D., King F.J., DeAngelo D.J., Partridge J.B., Holden S.A., Chen L.B., Singer S., Fletcher C. (1998). Differentiation and reversal of malignant changes in colon cancer through PPARgamma. Nat. Med..

[169] Hase T., Yoshimura R., Mitsuhashi M., Segawa Y., Kawahito Y., Wada S., Nakatani T., Sano H. (2002). Expression of peroxisome proliferator-activated receptors in human testicular cancer and growth inhibition by its agonists. Urology.

[170] Wang X., Wang G., Shi Y., Sun L., Gorczynski R., Li Y.J., Xu Z., Spaner D.E. (2016). PPAR-delta promotes survival of breast cancer cells in harsh metabolic conditions. Oncogenesis.

[171] Brockman J.A., Gupta R.A., DuBois R.N. (1998). Activation of PPARγ leads to inhibition of anchorage-independent growth of human colorectal cancer cells. Gastroenterology.

[172] Michalik L., Desvergne B., Wahli W. (2004). Peroxisome-proliferator-activated receptors and cancers: Complex stories. Nat. Nat. Rev. Cancer.

[173] Tanaka T., Kohno H., Yoshitani S.I., Takashima S., Okumura A., Murakami A., Hosokawa M. (2001). Ligands for peroxisome proliferator-activated receptors alpha and gamma inhibit chemically induced colitis and formation of aberrant crypt foci in rats. Cancer Res..

[174] Osawa E., Nakajima A., Wada K., Ishimine S., Fujisawa N., Kawamori T., Matsuhashi N., Kadowaki T., Ochiai M., Sekihara H. (2003). Peroxisome proliferator-activated receptor gamma ligands suppress colon carcinogenesis induced by azoxymethane in mice. Gastroenterology.

[175] Saez E., Tontonoz P., Nelson M.C., Alvarez J.G., Baird S.M., Thomazy V.A., Evans R.M. (1998). Activators of the nuclear receptor PPARgamma enhance colon polyp formation. Nat. Med..

[176] Lefebvre A.M., Chen I., Desreumaux P., Najib J., Fruchart J.C., Geboes K., Briggs M., Heyman R., Auwerx J. (1998). Activation of the peroxisome proliferator-activated receptor gamma promotes the development of colon tumors in C57BL/6J-APCMin/+ mice. Nat. Med..

[177] Wasan H.S., Novelli M., Bee J., Bodmer W.F. (1997). Dietary fat influences on polyp phenotype in multiple intestinal neoplasia mice. Proc. Natl. Acad. Sci. USA.

[178] Peters J.M., Hollingshead H.E., Gonzalez F.J. (2008). Role of peroxisome-proliferator-activated receptor beta/delta (PPARbeta/delta) in gastrointestinal tract function and disease. Clin. Sci..

[179] Peters J.M., Aoyama T., Cattley R.C., Nobumitsu U., Hashimoto T., Gonzalez F.J. (1998). Role of peroxisome proliferator-activated receptor alpha in altered cell cycle regulation in mouse liver. Carcinogenesis.

[180] Reed K.R., Sansom O.J., Hayes A.J., Gescher A.J., Winton D.J., Peters J.M., Clarke A.R. (2004). PPARδ status and Apc-mediated tumourigenesis in the mouse intestine. Oncogene.

[181] Shureiqi I., Jiang W., Zuo X., Wu Y., Stimmel J.B., Leesnitzer L.M., Morris J.S., Fan H.Z., Fischer S.M., Lippman S.M. (2003). The 15-lipoxygenase-1 product 13-S-hydroxyoctadecadienoic acid down-regulates PPAR-δ to induce apoptosis in colorectal cancer cells. Proc. Natl. Acad. Sci. USA.

[182] Brash A.R., Boeglin W.E., Chang M.S. (1997). Discovery of a second 15S-lipoxygenase in humans. Proc. Natl. Acad. Sci. USA.

[183] Baer A.N., Costello P.B., Green F.A. (1991). In vivo activation of an omega-6 oxygenase in human skin. Biochem. Biophys. Res. Commun..

[184] Ikawa H., Kamitani H., Calvo B.F., Foley J.F., Eling T.E. (1999). Expression of 15-lipoxygenase-1 in human colorectal cancer. Cancer Res..

[185] Shureiqi I., Chen D., Lotan R., Yang P., Newman R.A., Fischer S.M., Lippman S.M. (2000). 15-Lipoxygenase-1 mediates nonsteroidal anti-inflammatory drug-induced apoptosis independently of cyclooxygenase-2 in colon cancer cells. Cancer Res..

[186] Tachibana K., Yamasaki D., Ishimoto K. (2008). The Role of PPARs in Cancer. PPAR Res..

[187] Kim E.K., Choi E.-J. (2010). Pathological roles of MAPK signaling pathways in human diseases. Biochim. Biophys. Acta (Bba)–Mol. Basis Dis..

[188] Raman M., Chen W., Cobb M.H. (2007). Differential regulation and properties of MAPKs. Oncogene.

[189] Cargnello M., Roux P.P. (2011). Activation and function of the MAPKs and their substrates the MAPK-activated protein kinases. Microbiol. Mol. Biol. Rev..

[190] Dhillon A.S., Hagan S., Rath O., Kolch W. (2007). MAP kinase signalling pathways in cancer. Oncogene.

[191] Thalhamer T., McGrath M.A., Harnett M.M. (2008). MAPKs and their relevance to arthritis and inflammation. Rheumatology.

[192] Yang Y., Kim S.C., Yu T., Yi Y.S., Rhee M.H., Sung G.H., Yoo B.C., Cho J.Y. (2014). Functional roles of p38 mitogen-activated protein kinase in macrophage-mediated inflammatory responses. Mediat. Inflamm..

[193] Huang P., Han J., Hui L. (2010). MAPK signaling in inflammation-associated cancer development. Protein Cell Cell.

[194] Chen Y., Zhang Y., Chen S., Liu W., Lin Y., Zhang H., Yu F. (2021). Non-Steroidal Anti-Inflammatory Drugs (NSAIDs) sensitize melanoma cells to MEK inhibition and inhibit metastasis and relapse by inducing degradation of AXL. Pigment Cell Melanoma Res..

[195] Torii S., Yamamoto T., Tsuchiya Y., Nishida E. (2006). ERK MAP kinase in G1 cell cycle progression and cancer. Cancer Sci..

[196] Papachristou D.J., Batistatou A., Sykiotis G.P., Varakis I., Papavassiliou A.G. (2003). Activation of the JNK–AP-1 signal transduction pathway is associated with pathogenesis and progression of human osteosarcomas. Bone.

[197] Avisetti D.R., Babu K.S., Kalivendi S.V. (2014). Activation of p38/JNK pathway is responsible for embelin induced apoptosis in lung cancer cells: Transitional role of reactive oxygen species. PLoS ONE.

[198] Hardwick J.C.H., Van Den Brink G.R., Offerhaus G.J., Van Deventer S.J., Peppelenbosch M.P. (2001). NF-kappaB p38 MAPK and JNK are highly expressed and active in the stroma of human colonic adenomatous polyps. Oncogene.

[199] Malumbres M., Barbacid M. (2003). RAS oncogenes: The first 30 years. Nat. Nat. Rev. Cancer.

[200] Haigis K.M., Kendall K.R., Wang Y., Cheung A., Haigis M.C., Glickman J.N., Niwa-Kawakita M., Sweet-Cordero A., Sebolt-Leopold J., Shannon K.M. (2008). Differential effects of oncogenic K-Ras and N-Ras on proliferation differentiation and tumor progression in the colon. Nat. Genet..

[201] Bos J.L., Fearon E.R., Hamilton S.R., Verlaan–de Vries M., van Boom J.H., van der Eb A.J., Vogelstein B. (1987). Prevalence of ras gene mutations in human colorectal cancers. Nature.

[202] Forrester K., Almoguera C., Han K., Grizzle W.E., Perucho M. (1987). Detection of high incidence of K-ras oncogenes during human colon tumorigenesis. Nature.

[203] Rodenhuis S., van de Wetering M.L., Mooi W.J., Evers S.G., van Zandwijk N., Bos J.L. (1987). Mutational Activation of the K-ras Oncogene. N. Engl. J. Med..

[204] Almoguera C., Shibata D., Forrester K., Martin J., Arnheim N., Perucho M. (1988). Most human carcinomas of the exocrine pancreas contain mutant c-K-ras genes. Cell.

[205] Campbell S.L., Khosravi-Far R., Rossman K.L., Clark G.J., Der C.J. (1998). Increasing complexity of Ras signaling. Oncogene.

[206] Vogelstein B., Fearon E.R., Hamilton S.R., Kern S.E., Preisinger A.C., Leppert M., Smits A.M., Bos J.L. (1988). Genetic alterations during colorectal-tumor development. N. Engl. J. Med..

[207] Calcagno S.R., Li S., Colon M., Kreinest P.A., Thompson E.A., Fields A.P., Murray N.R. (2008). Oncogenic K-ras promotes early carcinogenesis in the mouse proximal colon. Int. J. Cancer.

[208] Schwenger P., Bellosta P., Vietor I., Basilico C., Skolnik E.Y., Vilček J. (1997). Sodium salicylate induces apoptosis via p38 mitogen-activated protein kinase but inhibits tumor necrosis factor-induced c-Jun N-terminal kinase/stress-activated protein kinase activation. Proc. Natl. Acad. Sci. USA.

[209] Schwenger P., Alpert D., Skolnik E.Y., Vilček J. (1999). Cell-type-specific activation of c-Jun N-terminal kinase by salicylates. J. Cell. Physiol..

[210] Yuan Z., Zhao J., Wang Z., Ren G., Zhang Z., Ma G. (2020). Effects of aspirin on hepatocellular carcinoma and its potential molecular mechanism. J. Buon.

[211] Sun J., Guo C., Zheng W., Zhang X. (2019). Aspirin inhibits proliferation and promotes apoptosis of hepatocellular carcinoma *Cells* via wnt/β-catenin signaling pathway. Panminerva Med..

[212] Li X., Xiang Y., Li F., Yin C., Li B., Ke X. (2019). WNT/β-catenin signaling pathway regulating t cell-inflammation in the tumor microenvironment. Front. Immunol..

[213] Silva-García O., Valdez-Alarcón J.J., Baizabal-Aguirre V.M. (2014). The Wntβ-Catenin signaling pathway controls the inflammatory response in infections caused by pathogenic bacteria. Mediat. Inflamm..

[214] Doucas H., Garcea G., Neal C.P., Manson M.M., Berry D.P. (2005). Changes in the Wnt signalling pathway in gastrointestinal cancers and their prognostic significance. Eur. J. Cancer.

[215] Pai S.G., Carneiro B.A., Mota J.M., Costa R., Leite C.A., Barroso-Sousa R., Kaplan J.B., Chae Y.K., Giles F.J. (2017). Wnt/beta-catenin pathway: Modulating anticancer immune response. J. Hematol. Oncol..

[216] Suryawanshi A., Tadagavadi R.K., Swafford D., Manicassamy S. (2016). Modulation of inflammatory responses by wnt/β-catenin signaling in dendritic cells: A novel immunotherapy target for autoimmunity and cancer. Front. Immunol..

[217] Ma B., Hottiger M.O. (2016). Crosstalk between Wnt/β-Catenin and NF-κB Signaling Pathway during Inflammation. Front. Immunol..

[218] Manoharan I., Hong Y., Suryawanshi A., Angus-Hill M.L., Sun Z., Mellor A.L., Munn D.H., Manicassamy S. (2014). TLR2-dependent activation of β-catenin pathway in dendritic cells induces regulatory responses and attenuates autoimmune inflammation. J. Immunol..

[219] Hadjihannas M.V., Brückner M., Jerchow B., Birchmeier W., Dietmaier W., Behrens J. (2006). Aberrant Wnt/β-catenin signaling can induce chromosomal instability in colon cancer. Proc. Natl. Acad. Sci. USA.

[220] Jung Y.-S., Jun S., Lee S.H., Sharma A., Park J.I. (2015). Wnt2 complements Wnt/β-catenin signaling in colorectal cancer. Oncotarget.

[221] Ying J., Li H., Yu J., Ng K.M., Poon F.F., Wong S.C.C., Chan A.T., Sung J.J., Tao Q. (2008). wnt5a exhibits tumor-suppressive activity through antagonizing the Wnt/β-catenin signaling and is frequently methylated in colorectal cancer. Clin. Cancer Res..

[222] Cai J., Guan H., Fang L., Yang Y., Zhu X., Yuan J., Wu J., Li M. (2013). MicroRNA-374a activates Wnt/β-catenin signaling to promote breast cancer metastasis. J. Clin. Investig..

[223] King T.D., Suto M.J., Li Y. (2012). The wnt/β-catenin signaling pathway: A potential therapeutic target in the treatment of triple negative breast cancer. J. Cell. Biochem..

[224] Jang G.-B., Kim J.Y., Cho S.D., Park K.S., Jung J.Y., Lee H.Y., Hong I.S., Nam J.S. (2015). Blockade of Wnt/β-catenin signaling suppresses breast cancer metastasis by inhibiting CSC-like phenotype. Sci. Rep..

[225] Lucijanic M., Livun A., Tomasovic-Loncaric C., Stoos-Veic T., Pejsa V., Jaksic O., Prka Z., Kusec R. (2016). Canonical Wnt/β-catenin signaling pathway is dysregulated in patients with primary and secondary myelofibrosis. Clin. Lymphoma Myeloma Leuk..

[226] Mathur R., Sehgal L., Braun F.K., Berkova Z., Romaguerra J., Wang M., Rodriguez M.A., Fayad L., Neelapu S.S., Samaniego F. (2015). Targeting Wnt pathway in mantle cell lymphoma-initiating cells. J. Hematol. Oncol..

[227] Geduk A., Atesoglu E.B., Tarkun P., Mehtap O., Hacihanefioglu A., Demirsoy E.T., Baydemir C. (2015). The Role of β-catenin in Bcr/Abl negative myeloproliferative neoplasms: An immunohistochemical study. Clin. Lymphoma Myeloma Leuk..

[228] Hong Y., Manoharan I., Suryawanshi A., Majumdar T., Angus-Hill M.L., Koni P.A., Manicassamy B., Mellor A.L., Munn D.H., Manicassamy S. (2015). β-catenin promotes regulatory T-cell responses in tumors by inducing vitamin a metabolism in dendritic cells. Cancer Res..

[229] Damsky William E., Damsky W.E., Curley D.P., Santhanakrishnan M., Rosenbaum L.E., Platt J.T., Rothberg B.E.G., Taketo M.M., Dankort D., Rimm D.L. (2011). β-Catenin signaling controls metastasis in Braf-activated Pten-deficient melanomas. Cancer Cell.

[230] Gallagher S.J., Rambow F., Kumasaka M., Champeval D., Bellacosa A., Delmas V., Larue L. (2013). Beta-catenin inhibits melanocyte migration but induces melanoma metastasis. Oncogene.

[231] Hoseong Yang S., Andl T., Grachtchouk V., Wang A., Liu J., Syu L.J., Ferris J., Wang T.S., Glick A.B., Millar S.E. (2008). Pathological responses to oncogenic Hedgehog signaling in skin are dependent on canonical Wnt/β-catenin signaling. Nat. Genet..

[232] Vallée A., Lecarpentier Y., Vallée J.N. (2019). Targeting the canonical WNT/β-catenin pathway in cancer treatment using non-steroidal anti-inflammatory drugs. Cells.

[233] Akrami H., Moradi B., Borzabadi Farahani D., Mehdizadeh K. (2018). Ibuprofen reduces *Cell* Prolif.eration through inhibiting Wnt/β catenin signaling pathway in gastric cancer stem cells. Cell Biol. Int..

[234] Azevedo M.F., Faucz F.R., Bimpaki E., Horvath A., Levy I., de Alexandre R.B., Ahmad F., Manganiello V., Stratakis C.A. (2014). Clinical and molecular genetics of the phosphodiesterases (PDEs). Endocr. Rev..

[235] Yamanaka Y., Mammoto T., Kirita T., Mukai M., Mashimo T., Sugimura M., Kishi Y., Nakamura H. (2002). Epinephrine inhibits invasion of oral squamous carcinoma cells by modulating intracellular cAMP. Cancer Lett..

[236] Hirsh L., Dantes A., Suh B.S., Yoshida Y., Hosokawa K., Tajima K., Kotsuji F., Merimsky O., Amsterdam A. (2004). Phosphodiesterase inhibitors as anti-cancer drugs. Biochem. Pharm..

[237] Sarfati M., Mateo V., Baudet S., Rubio M., Fernandez C., Davi F., Binet J.L., Delic J., Merle-Béral H. (2003). Sildenafil and vardenafil types 5 and 6 phosphodiesterase inhibitors induce caspase-dependent apoptosis of B-chronic lymphocytic leukemia cells. Blood.

[238] Li N., Lee K., Xi Y., Zhu B., Gary B.D., Ramírez-Alcántara V., Gurpinar E., Canzoneri J.C., Fajardo A., Sigler S. (2015). Phosphodiesterase 10A: A novel target for selective inhibition of colon tumor cell growth and β-catenin-dependent TCF transcriptional activity. Oncogene.

[239] Piazza G.A., Ward A., Chen X., Maxuitenko Y., Coley A., Aboelella N.S., Buchsbaum D.J., Boyd M.R., Keeton A.B., Zhou G. (2020). PDE5 and PDE10 inhibition activates cGMP/PKG signaling to block Wnt/β-catenin transcription cancer cell growth and tumor immunity. Drug Discov. Today.

[240] Zhu B., Lindsey A., Li N., Lee K., Ramirez-Alcantara V., Canzoneri J.C., Fajardo A., da Silva L.M., Thomas M., Piazza J.T. (2017). Phosphodiesterase 10A is overexpressed in lung tumor cells and inhibitors selectively suppress growth by blocking β-catenin and MAPK signaling. Oncotarget.

[241] Wullschleger S., Loewith R., Hall M.N. (2006). TOR Signaling in growth and metabolism. Cell.

[242] Zou Z., Tao T., Li H., Zhu X. (2020). mTOR signaling pathway and mTOR inhibitors in cancer: Progress and challenges. Cell Biosci..

[243] Lim H.J., Crowe P., Yang J.L. (2015). Current clinical regulation of PI3K/PTEN/Akt/mTOR signalling in treatment of human cancer. J. Cancer Res. Clin. Oncol..

[244] Ricoult S.J., Yecies J.L., Ben-Sahra I., Manning B.D. (2016). Oncogenic PI3K and K-Ras stimulate de novo lipid synthesis through mTORC1 and SREBP. Oncogene.

[245] Chen Y., Qian J., He Q., Zhao H., Toral-Barza L., Shi C., Zhang X., Wu J., Yu K. (2016). mTOR complex-2 stimulates acetyl-CoA and de novo lipogenesis through ATP citrate lyase in HER2/PIK3CA-hyperactive breast cancer. Oncotarget.

[246] Hsieh A.C., Liu Y., Edlind M.P., Ingolia N.T., Janes M.R., Sher A., Shi E.Y., Stumpf C.R., Christensen C., Bonham M.J. (2012). The translational landscape of mTOR signalling steers cancer initiation and metastasis. Nature.

[247] Guri Y., Colombi M., Dazert E., Hindupur S.K., Roszik J., Moes S., Jenoe P., Heim M.H., Riezman I., Riezman H. (2017). mTORC2 Promotes Tumorigenesis via Lipid Synthesis. Cancer Cell.

[248] Di Malta C., Siciliano D., Calcagni A., Monfregola J., Punzi S., Pastore N., Eastes A.N., Davis O., De Cegli R., Zampelli A. (2017). Transcriptional activation of RagD GTPase controls mTORC1 and promotes cancer growth. Science.

[249] Ogier-Denis E., Codogno P. (2003). Autophagy: A barrier or an adaptive response to cancer. Biochim. Biophys. Acta (Bba)—Rev. Cancer.

[250] Kim J., Klionsky D.J. (2000). Autophagy cytoplasm-to-vacuole targeting pathway and pexophagy in yeast and mammalian cells. Annu. Rev. Biochem..

[251] Kanzawa T., Germano I.M., Komata T., Ito H., Kondo Y., Kondo S. (2004). Role of autophagy in temozolomide-induced cytotoxicity for malignant glioma cells. Cell Death Differ.

[252] Dickstein R.J., Nitti G., Dinney C.P., Davies B.R., Kamat A.M., McConkey D.J. (2012). Autophagy limits the cytotoxic effects of the AKT inhibitor AZ7328 in human bladder cancer cells. Cancer Biol. Ther..

[253] Paglin S., Hollister T., Delohery T., Hackett N., McMahill M., Sphicas E., Domingo D., Yahalom J. (2001). A novel response of cancer cells to radiation involves autophagy and formation of acidic vesicles. Cancer Res..

[254] Yu C., Li W.B., Liu J.B., Lu J.W., Feng J.F. (2018). Autophagy: Novel applications of nonsteroidal anti-inflammatory drugs for primary cancer. Cancer Med..

[255] Lu Z., Luo R.Z., Lu Y., Zhang X., Yu Q., Khare S., Kondo S., Kondo Y., Yu Y., Mills G.B. (2008). The tumor suppressor gene ARHI regulates autophagy and tumor dormancy in human ovarian cancer cells. J. Clin. Investig..

[256] Butler D.E., Marlein C., Walker H.F., Frame F.M., Mann V.M., Simms M.S., Davies B.R., Collins A.T., Maitland N.J. (2017). Inhibition of the PI3K/AKT/mTOR pathway activates autophagy and compensatory Ras/Raf/MEK/ERK signalling in prostate cancer. Oncotarget.

[257] Kim K.W., Mutter R.W., Cao C., Albert J.M., Freeman M., Hallahan D.E., Lu B. (2006). Autophagy for cancer therapy through inhibition of pro-apoptotic proteins and mammalian target of rapamycin signaling. J. Biol. Chem..

[258] Cory S., Adams J.M. (2002). The Bcl2 family: Regulators of the cellular life-or-death switch. Nat. Rev. Cancer.

[259] Green D.R., Reed J.C. (1998). Mitochondria and apoptosis. Science.

[260] Jürgensmeier J.M., Xie Z., Deveraux Q., Ellerby L., Bredesen D., Reed J.C. (1998). Bax directly induces release of cytochrome c from isolated mitochondria. Proc. Natl. Acad. Sci. USA.

[261] Cai J., Yang J., Jones D. (1998). Mitochondrial control of apoptosis: The role of cytochrome c. Biochim. Biophys. Acta (Bba)—Bioenerg..

[262] Zhivotovsky B., Orrenius S., Brustugun O.T., Døskeland S.O. (1998). Injected cytochrome c induces apoptosis. Nature.

[263] Wang X., Baek S.J., Eling T.E. (2013). The diverse roles of nonsteroidal anti-inflammatory drug activated gene (NAG-1/GDF15) in cancer. Biochem. Pharm..

[264] Martinez J.M., Sali T., Okazaki R., Anna C., Hollingshead M., Hose C., Monks A., Walker N.J., Baek S.J., Eling T.E. (2006). Drug-induced expression of nonsteroidal anti-inflammatory drug-activated gene/macrophage inhibitory cytokine-1/prostate-derived factor a putative tumor suppressor inhibits tumor growth. J. Pharm. Exp. Therap..

[265] Kawahara T., Ishiguro H., Hoshino K., Teranishi J.I., Miyoshi Y., Kubota Y., Uemura H. (2010). Analysis of NSAID-activated gene 1 expression in prostate cancer. Urol. Int..

[266] Monteith G.R., McAndrew D., Faddy H.M., Roberts-Thomson S.J. (2007). Calcium and cancer: Targeting Ca^2+^ transport. Nat. Rev. Cancer.

[267] Panda S., Chatterjee O., Roy L., Chatterjee S. (2021). Targeting Ca^2+^ signaling: A new arsenal against cancer. Drug Discov. Today.

[268] Ibrahim S., Dakik H., Vandier C., Chautard R., Paintaud G., Mazurier F., Lecomte T., Guéguinou M., Raoul W. (2019). Expression profiling of calcium channels and calcium-activated potassium channels in colorectal cancer. Cancers.

[269] Phan N.N., Wang C.Y., Chen C.F., Sun Z., Lai M.D., Lin Y.C. (2017). Voltage-gated calcium channels: Novel targets for cancer therapy. Oncol. Lett..

[270] Sritangos P., Pena Alarcon E., James A.D., Sultan A., Richardson D.A., Bruce J.I. (2020). Plasma Membrane Ca(2+) ATPase isoform 4 (PMCA4) has an important role in numerous hallmarks of pancreatic cancer. Cancers.

[271] Silvestri R., Pucci P., Venalainen E., Matheou C., Mather R., Chandler S., Aceto R., Rigas S.H., Wang Y., Rietdorf K. (2019). T-type calcium channels drive the proliferation of androgen-receptor negative prostate cancer cells. Prostate.

[272] Das A., Pushparaj C., Bahí N., Sorolla A., Herreros J., Pamplona R., Vilella R., Matias-Guiu X., Marti R.M., Cantí C. (2012). Functional expression of voltage-gated calcium channels in human melanoma. Pigment Cell Melanoma Res..

[273] Cabanas H., Harnois T., Magaud C., Cousin L., Constantin B., Bourmeyster N., Déliot N. (2018). Deregulation of calcium homeostasis in Bcr-Abl-dependent chronic myeloid leukemia. Oncotarget.

[274] Nishida N., Yano H., Nishida T., Kamura T., Kojiro M. (2006). Angiogenesis in cancer. Vasc. Health Risk Manag..

[275] Kerbel R.S. (2008). Tumor Angiogenesis. N. Engl. J. Med..

[276] Holmgren L., O’Reilly M.S., Folkman J. (1995). Dormancy of micrometastases: Balanced proliferation and apoptosis in the presence of angiogenesis suppression. Nat. Med..

[277] Streit M., Riccardi L., Velasco P., Brown L.F., Hawighorst T., Bornstein P., Detmar M. (1999). Thrombospondin-2: A potent endogenous inhibitor of tumor growth and angiogenesis. Proc. Natl. Acad. Sci. USA.

[278] Dews M., Homayouni A., Yu D., Murphy D., Sevignani C., Wentzel E., Furth E.E., Lee W.M., Enders G.H., Mendell J.T. (2006). Augmentation of tumor angiogenesis by a Myc-activated microRNA cluster. Nat. Genet..

[279] Tiwari M. (2012). Apoptosis angiogenesis and cancer therapies. J. Cancer Ther. Res..

[280] Tarnawski A.S., Jones M.K. (2003). Inhibition of angiogenesis by NSAIDs: Molecular mechanisms and clinical implications. J. Mol. Med..

[281] Pastorekova S., Parkkila S., Pastorek J., Supuran C.T. (2004). Carbonic anhydrases: Current state of the art, therapeutic applications and future prospects. J. Enzym. Inhib. Med. Chem..

[282] Chiche J., Ilc K., Laferriere J., Trottier E., Dayan F., Mazure N.M., Brahimi-Horn M.C., Pouysségur J. (2009). Hypoxia-inducible carbonic anhydrase IX and XII promote tumor cell growth by counteracting acidosis through the regulation of the intracellular pH. Cancer Res..

[283] Singh S., Lomelino C.L., Mboge M.Y., Frost S.C., McKenna R. (2018). Cancer drug development of carbonic anhydrase inhibitors beyond the active site. Molecules.

[284] Pastorek J., Pastorekova S. (2015). Hypoxia-induced carbonic anhydrase IX as a target for cancer therapy: From biology to clinical use. Semin. Cancer Biol..

[285] Hynninen P., Vaskivuo L., Saarnio J., Haapasalo H., Kivelä J., Pastorekova S., Pastorek J., Waheed A., Sly W.S., Puistola U. (2006). Expression of transmembrane carbonic anhydrases IX and XII in ovarian tumours. Histopathology.

[286] Kim J.Y., Shin H.J., Kim T.H., Cho K.H., Shin K.H., Kim B.K., Roh J.W., Lee S., Park S.Y., Hwang Y.J. (2006). Tumor-associated carbonic anhydrases are linked to metastases in primary cervical cancer. J. Cancer Res. Clin. Oncol..

[287] Tafreshi N.K., Bui M.M., Bishop K., Lloyd M.C., Enkemann S.A., Lopez A.S., Abrahams D., Carter B.W., Vagner J., Grobmyer S.R. (2012). Noninvasive Detection of Breast Cancer Lymph Node Metastasis Using Carbonic Anhydrases IX and XII Targeted Imaging Probes. Clin. Cancer Res..

[288] Hussain S.A., Ganesan R., Reynolds G., Gross L., Stevens A., Pastorek J., Murray P.G., Perunovic B., Anwar M.S., Billingham L. (2007). Hypoxia-regulated carbonic anhydrase IX expression is associated with poor survival in patients with invasive breast cancer. Br. J. Cancer.

[289] Kivelä A.J., Parkkila S., Saarnio J., Karttunen T.J., Kivelä J., Parkkila A.K., Pastoreková S., Pastorek J., Waheed A., Sly W.S. (2000). Expression of transmembrane carbonic anhydrase isoenzymes IX and XII in normal human pancreas and pancreatic tumours. Histochem. Cell Biol..

[290] Järvelä S., Parkkila S., Bragge H., Kähkönen M., Parkkila A.K., Soini Y., Pastorekova S., Pastorek J., Haapasalo H. (2008). Carbonic anhydrase IX in oligodendroglial brain tumors. BMC Cancer.

[291] Buckley C.D., Gilroy D.W., Serhan C.N. (2014). Serhan Proresolving Lipid Mediators and Mechanisms in the Resolution of Acute Inflammation. Immunity.

[292] Janakiram N.B., Mohammed A., Rao C.V. (2011). Role of lipoxins resolvins and other bioactive lipids in colon and pancreatic cancer. Cancer Metastasis Rev..

[293] Weylandt K.H., Chiu C.Y., Gomolka B., Waechter S.F., Wiedenmann B. (2012). Omega-3 fatty acids and their lipid mediators: Towards an understanding of resolvin and protectin formation. Prostaglandins Other Lipid Mediat..

[294] Panigrahy D., Gilligan M.M., Serhan C.N., Kashfi K. (2021). Resolution of inflammation: An organizing principle in biology and medicine. Pharm. Ther..

[295] Serhan C.N., Petasis N.A. (2011). Resolvins and protectins in inflammation resolution. Chem. Rev..

[296] Serhan C.N., Hamberg M., Samuelsson B. (1984). Lipoxins: Novel series of biologically active compounds formed from arachidonic acid in human leukocytes. Proc. Natl. Acad. Sci. USA.

[297] Serhan C.N., Dalli J., Colas R.A., Winkler J.W., Chiang N. (2015). Protectins and maresins: New pro-resolving families of mediators in acute inflammation and resolution bioactive metabolome. Biochim. Biophys. Acta (Bba)—Mol. Cell Biol. Lipids.

[298] Serhan C.N. (2014). Pro-resolving lipid mediators are leads for resolution physiology. Nature.

[299] Berquin I.M., Edwards I.J., Chen Y.Q. (2008). Multi-targeted therapy of cancer by omega-3 fatty acids. Cancer Lett..

[300] Ding Y., Mullapudi B., Torres C., Mascariñas E., Mancinelli G., Diaz A.M., McKinney R., Barron M., Schultz M., Heiferman M. (2018). Omega-3 fatty acids prevent early pancreatic carcinogenesis via repression of the AKT pathway. Nutrients.

[301] Nowak J., Weylandt K.H., Habbel P., Wang J., Dignass A., Glickman J.N., Kang J.X. (2007). Colitis-associated colon tumorigenesis is suppressed in transgenic mice rich in endogenous n-3 fatty acids. Carcinogenesis.

[302] Weylandt K.H., Krause L.F., Gomolka B., Chiu C.Y., Bilal S., Nadolny A., Waechter S.F., Fischer A., Rothe M., Kang J.X. (2011). Suppressed liver tumorigenesis in fat-1 mice with elevated omega-3 fatty acids is associated with increased omega-3 derived lipid mediators and reduced TNF-α. Carcinogenesis.

[303] Fishbein A., Hammock B.D., Serhan C.N., Panigrahy D. (2021). Carcinogenesis: Failure of resolution of inflammation?. Pharm. Ther..

[304] Filippou P.S., Karagiannis G.S. (2020). Cytokine storm during chemotherapy: A new companion diagnostic emerges?. Oncotarget.

[305] Hammock B.D. (2020). Eicosanoids: The overlooked storm in coronavirus disease 2019 (COVID-19)?. Am. J. Pathol..

[306] Panigrahy D., Gilligan M.M., Huang S., Gartung A., Cortés-Puch I., Sime P.J., Phipps R.P., Serhan C.N., Hammock B.D. (2020). Inflammation resolution: A dual-pronged approach to averting cytokine storms in COVID-19?. Cancer Metastasis Rev..

[307] Recchiuti A., Serhan C.N. (2012). Pro-resolving lipid mediators (SPMs) and their actions in regulating mirna in novel resolution circuits in inflammation. Front. Immunol..

[308] Levy B.D., Clish C.B., Schmidt B., Gronert K., Serhan C.N. (2001). Lipid mediator class switching during acute inflammation: Signals in resolution. Nat. Immunol..

[309] Petasis N.A., Akritopoulou-Zanze I., Fokin V.V., Bernasconi G., Keledjian R., Yang R., Uddin J., Nagulapalli K.C., Serhan C.N. (2005). Design synthesis and bioactions of novel stable mimetics of lipoxins and aspirin-triggered lipoxins. Prostaglandins Leukot. Essent. Fat. Acids.

[310] Romano M. (2010). Lipoxin and aspirin-triggered lipoxins. ScientificWorldJournal.

[311] Clària J., Serhan C.N. (1995). Aspirin triggers previously undescribed bioactive eicosanoids by human endothelial cell-leukocyte interactions. Proc. Natl. Acad. Sci. USA.

[312] Colgan S.P., Colgan S.P., Serhan C.N., Parkos C.A., Delp-Archer C., Madara J.L. (1993). Lipoxin A4 modulates transmigration of human neutrophils across intestinal epithelial monolayers. J. Clin. Investig..

[313] Oh S.F., Pillai P.S., Recchiuti A., Yang R., Serhan C.N. (2011). Pro-resolving actions and stereoselective biosynthesis of 18S E-series resolvins in human leukocytes and murine inflammation. J. Clin. Investig..

[314] Capdevila J.H., Wei S., Helvig C., Falck J.R., Belosludtsev Y., Truan G., Graham-Lorence S.E., Peterson J.A. (1996). The highly stereoselective oxidation of polyunsaturated fatty acids by cytochrome P450BM-3. J. Biol. Chem..

[315] Tjonahen E., Oh S.F., Siegelman J., Elangovan S., Percarpio K.B., Hong S., Arita M., Serhan C.N. (2006). Resolvin E2: Identification and anti-inflammatory actions: Pivotal role of human 5-lipoxygenase in resolvin E series biosynthesis. Chem. Biol..

[316] Serhan C.N., Clish C.B., Brannon J., Colgan S.P., Chiang N., Gronert K. (2000). Novel functional sets of lipid-derived mediators with antiinflammatory actions generated from omega-3 fatty acids via cyclooxygenase 2-nonsteroidal antiinflammatory drugs and transcellular processing. J. Exp. Med..

[317] Serhan C.N., Hong S., Gronert K., Colgan S.P., Devchand P.R., Mirick G., Moussignac R.L. (2002). Resolvins: A Family of bioactive products of omega-3 fatty acid transformation circuits initiated by aspirin treatment that counter proinflammation signals. J. Exp. Med..

[318] Sun Y.P., Oh S.F., Uddin J., Yang R., Gotlinger K., Campbell E., Colgan S.P., Petasis N.A., Serhan C.N. (2007). Resolvin D1 and its aspirin-triggered 17R epimer. Stereochemical assignments anti-inflammatory properties and enzymatic inactivation. J. Biol. Chem..

[319] Dalli J., Chiang N., Serhan C.N. (2015). Elucidation of novel 13-series resolvins that increase with atorvastatin and clear infections. Nat. Med..

[320] Fullerton J.N., O’Brien A.J., Gilroy D.W. (2014). Lipid mediators in immune dysfunction after severe inflammation. Trends Immunol..

[321] Serhan C.N., Gotlinger K., Hong S., Lu Y., Siegelman J., Baer T., Yang R., Colgan S.P., Petasis N.A. (2006). Anti-inflammatory actions of neuroprotectin D1/Protectin D1 and its natural stereoisomers: Assignments of dihydroxy-containing docosatrienes. J. Immunol..

[322] Deng B., Wang C.W., Arnardottir H.H., Li Y., Cheng C.Y.C., Dalli J., Serhan C.N. (2014). Maresin Biosynthesis and identification of Maresin 2 a new anti-inflammatory and pro-resolving mediator from human macrophages. PLoS ONE.

[323] Serhan C.N., Yang R., Martinod K., Kasuga K., Pillai P.S., Porter T.F., Oh S.F., Spite M. (2009). Maresins: Novel macrophage mediators with potent antiinflammatory and proresolving actions. J. Exp. Med..

[324] Serhan Charles N., Fredman G., Yang R., Karamnov S., Belayev L.S., Bazan N.G., Zhu M., Winkler J.W., Petasis N.A. (2011). Novel proresolving aspirin-triggered DHA pathway. Chem. Biol..

[325] Dalli J., Zhu M., Vlasenko N.A., Deng B., Haeggström J.Z., Petasis N.A., Serhan C.N. (2013). The novel 13S,14S-epoxy-maresin is converted by human macrophages to maresin 1 (MaR1) inhibits leukotriene A4 hydrolase (LTA4H) and shifts macrophage phenotype. FASEB J..

[326] Serhan C.N., Dalli J., Karamnov S., Choi A., Park C.K., Xu Z.Z., Ji R.R., Zhu M., Petasis N.A. (2012). Macrophage proresolving mediator maresin 1 stimulates tissue regeneration and controls pain. FASEB J..

[327] Mudge D., Kieran M.W., Bielenberg D., Benny O., Dalli J., Huang S., Serhan C.N., Panigrahy D. Maresin 1: A potent endogenous anti-inflammatory and pro-resolving inhibitor of primary tumor growth and metastasis. Proceedings of the AACR Annual Meeting.

[328] Vatnick D.R., Lehner K., Gilligan M., Panigrahy D., Gus-Brautbar Y., Ramon S., Huang S., Serhan C. (2016). Control of breast cancer through the resolution of inflammation. FASEB J..

